# Quantitative Analyses of Foot Processes, Mitochondria, and Basement Membranes by Structured Illumination Microscopy Using Elastica-Masson– and Periodic-Acid-Schiff–Stained Kidney Sections

**DOI:** 10.1016/j.ekir.2021.04.021

**Published:** 2021-05-01

**Authors:** Ayumi Matsumoto, Isao Matsui, Yusuke Katsuma, Seiichi Yasuda, Karin Shimada, Tomoko Namba-Hamano, Yusuke Sakaguchi, Jun-ya Kaimori, Yoshitsugu Takabatake, Kazunori Inoue, Yoshitaka Isaka

**Affiliations:** 1 Department of Nephrology, Osaka University Graduate School of Medicine, Osaka, Japan; 2 Department of Inter-Organ Communication Research in Kidney Disease, Osaka University Graduate School of Medicine, Osaka, Japan

**Keywords:** basement membrane, foot process, Fourier transform, kidney biopsy, mitochondrion, structured illumination microscopy

## Abstract

**Introduction:**

Foot process effacement and mitochondrial fission associate with kidney disease pathogenesis. Electron microscopy is the gold-standard method for their visualization, but the observable area of electron microscopy is smaller than light microscopy. It is important to develop alternative ways to quantitatively evaluate these microstructural changes because the lesion site of renal diseases can be focal.

**Methods:**

We analyzed elastica-Masson trichrome (EMT) and periodic acid-Schiff (PAS) stained kidney sections using structured illumination microscopy (SIM).

**Results:**

EMT staining revealed three-dimensional (3D) structures of foot process, whereas ponceau xylidine acid fuchsin azophloxine solution induced fluorescence. Conversion of foot process images into their constituent frequencies by Fourier transform showed that the concentric square of (1/4)^2^-(1/16)^2^ in the power spectra (PS) included information for normal periodic structures of foot processes. Foot process integrity, assessed by PS, negatively correlated with proteinuria. EMT-stained sections revealed fragmented mitochondria in mice with mitochondrial injuries and patients with tubulointerstitial nephritis; Fourier transform quantified associated mitochondrial injury. Quantified mitochondrial damage in patients with immunoglobulin A (IgA) nephropathy predicted a decline in estimated glomerular filtration rate (eGFR) after kidney biopsy but did not correlate with eGFR at biopsy. PAS-stained sections, excited by a 640 nm laser, combined with the coefficient of variation values, quantified subtle changes in the basement membranes of patients with membranous nephropathy stage I.

**Conclusions:**

Kidney microstructures are quantified from sections prepared in clinical practice using SIM.

## Graphical abstract

**Figure:**
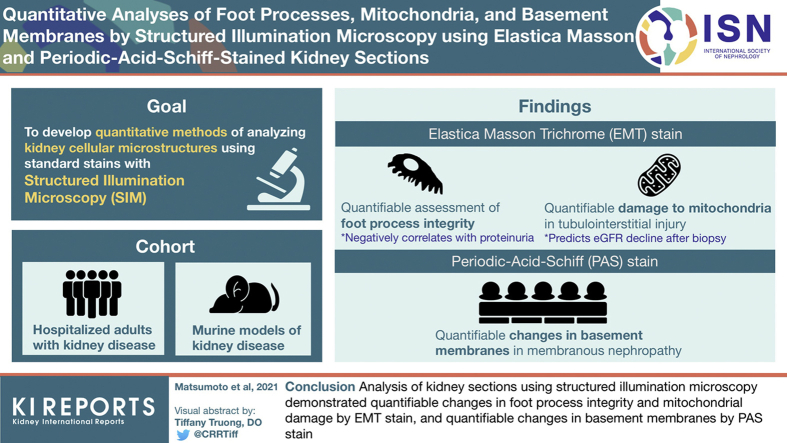

Various studies have shown that changes in cellular microstructures play important roles in the pathogenesis of renal diseases. For example, structural defects in podocyte foot processes can result in loss of the proper glomerular filtration barrier, whereas mitochondrial fragmentation can cause mitochondrial dysfunction and oxidative stress.^1^, ^2^, ^3^, ^4^, ^5^, ^6^ Electron microscopy is the gold-standard method for visualization of foot processes and mitochondria, but the observable area of electron microscopy is smaller than that for light microscopy because epon-embedded biopsy samples are usually smaller than paraffin-embedded samples. It is important to develop alternative ways to quantitatively evaluate these microstructural changes using paraffin-embedded samples because the lesion site of renal diseases can be focal.

The highest achievable resolution of a conventional optical microscope cannot surpass the diffraction limit (approximately 200–250 nm).^7^ However, various efforts have been made to surpass this limit. For example, re-scan confocal microscopy has achieved ~170-nm resolution, whereas conventional confocal microscopy provides ~240-nm resolution for 488-nm excitation.^8^ Confocal reflection microscopy has been reported to achieve ~30% resolution improvement in Golgi-Cox–stained specimens by minimizing the pinhole size to 0.1 airy unit.^9^ Furthermore, expansion microscopy has achieved 60-nm resolution in fluorescently labeled tissues.^10^, ^11^, ^12^, ^13^ In addition, super-resolution microscopy (SRM) also bypasses the diffraction limit and enables imaging of microstructures at a level of detail that was previously thought to be achievable only with electron microscopy.^7^^,^^14^ In fact, several types of SRM have been used for analysis of the podocyte foot process.^11^^,^^15^, ^16^, ^17^, ^18^ Unnersjö-Jess *et al.*^19^ reported that the podocin-stained slit diaphragm in optically cleared kidney tissues could be visualized by stimulated emission depletion microscopy; whereas, stochastic optical reconstruction microscopy has been used to analyze injury-induced actin cytoskeleton reorganization in podocytes.^16^ Meanwhile, SIM recently gained attention because it has advantages in reconstructing 3D images and is compatible with standard fluorescent dyes.^20^^,^^21^ Using SIM, Siegerist *et al.*^15^ have shown that a nephrin-stained slit diaphragm can be visualized.

Immunofluorescent staining is the standard sample preparation method for SRM analyses. However, antibodies are expensive. Organelle-specific fluorescent dyes are alternative tools for labeling cellular microstructures. For example, phalloidin, paclitaxel, and MitoTracker (Invitrogen, Waltham, MA) can label f-actin, tubulin, and mitochondria, respectively.^22^^,^^23^ However, paclitaxel and MitoTracker are not applicable to fixed cells, including paraffin-embedded kidney sections. Conventional tissue-staining dyes have also been reported to possess fluorescent properties. Apgar *et al.*^24^ has shown that eosinophilic structures in hematoxylin and eosin (HE)–stained paraffin-embedded sections were visualized using conventional fluorescent microscopy. Zhang *et al.*^25^ has shown that SIM can visualize the 3D structure of blood cells using Wright-Giemsa–stained blood smears. These studies prompted us to hypothesize that cellular microstructures in kidney biopsy samples can be visualized using SIM without specific organelle staining.

Here, we analyzed kidney cellular microstructures using SIM for HE-, PAS-, periodic acid methenamine silver (PAM)–, and EMT-stained paraffin-embedded sections prepared in daily clinical practice. Additionally, by applying Fourier transform (FT), we developed a method to quantify structural changes in the foot processes and mitochondria of kidney biopsy specimens.

## Methods

### Human Samples

**Figure 1:**
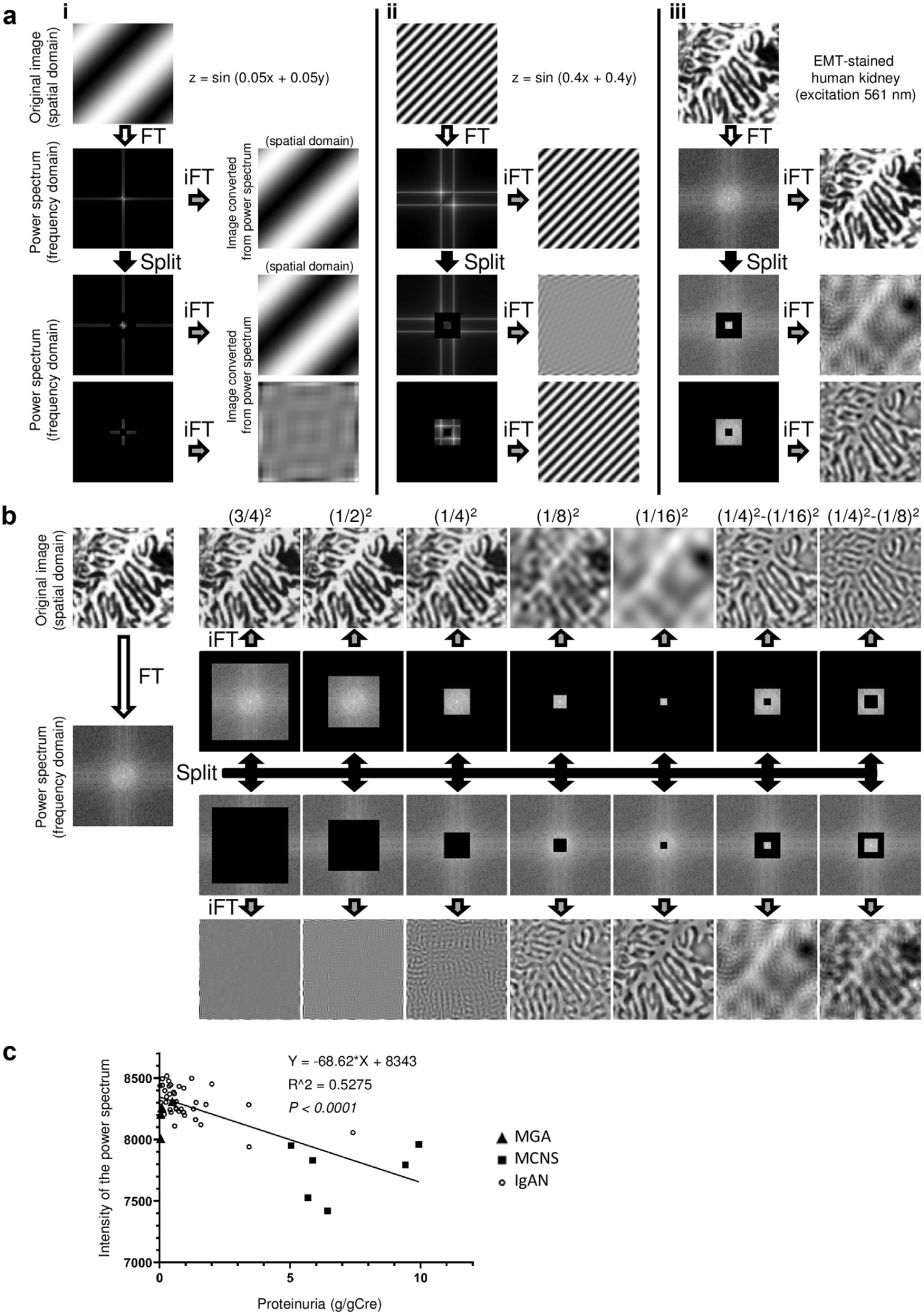
Quantification of foot process effacement using Fourier transform. (a) Analytical background for podocyte injury quantification is shown. Sine waves that express the following equations are visualized as 128 × 128 pixels black and white images: (i) z = sin (0.05x + 0.05y) and (ii) z = sin (0.4x + 0.4y). (iii) A representative podocyte foot process image (128 × 128 pixels) obtained from an elastica-Masson trichrome (EMT)–stained human kidney section that was excited at 561 nm. The sine wave in (i) mimics the periodicity of the podocyte primary process, whereas the sine wave in (ii) mimics the periodicity of foot process. Fourier transform (FT) decomposes an image (spatial domain) into its sine and cosine components (frequency domain). Power spectra (PS) are the result of FT and represent the image in the frequency domain, in which each point indicates strength of the particular frequency contained in the spatial domain image. The PS can be reconverted into the spatial domain image by inverse FT (iFT). The lower half of (a) shows how information in the PS affects the results of the iFT. The PS were split into a concentric square and its remainder. The sine wave observed in (i), but not in (ii) or the foot process in (iii), could be reconstructed from the remainder of the PS. Conversely, the sine wave shown in (ii) and foot process shown in (iii), but not sine wave shown in (i), could be reconstructed from the concentric square area. (b) The PS of the foot process image were split into various portions and reconstructed spatial domain images from the corresponding split PS are shown. A concentric square of (1/4)^2^-(1/16)^2^ adequately contained frequencies related to normal foot process structures. (c) Foot process three-dimensional structured illumination microscopy (3D SIM) images (128 × 128 pixels) from patients with minor glomerular abnormalities (MGAs), minimal change nephrotic syndrome (MCNS), and immunoglobulin A nephropathy (IgAN) were converted into PS. The PS intensity within the concentric square of (1/4)^2^-(1/16)^2^ was calculated and plotted on the vertical axis. The horizontal axis indicates urinary protein levels. A minimum of 10 images per patient were analyzed. (N = 48 patients, R^2^ = 0.528; *P* < 0.0001).

**Figure 2:**
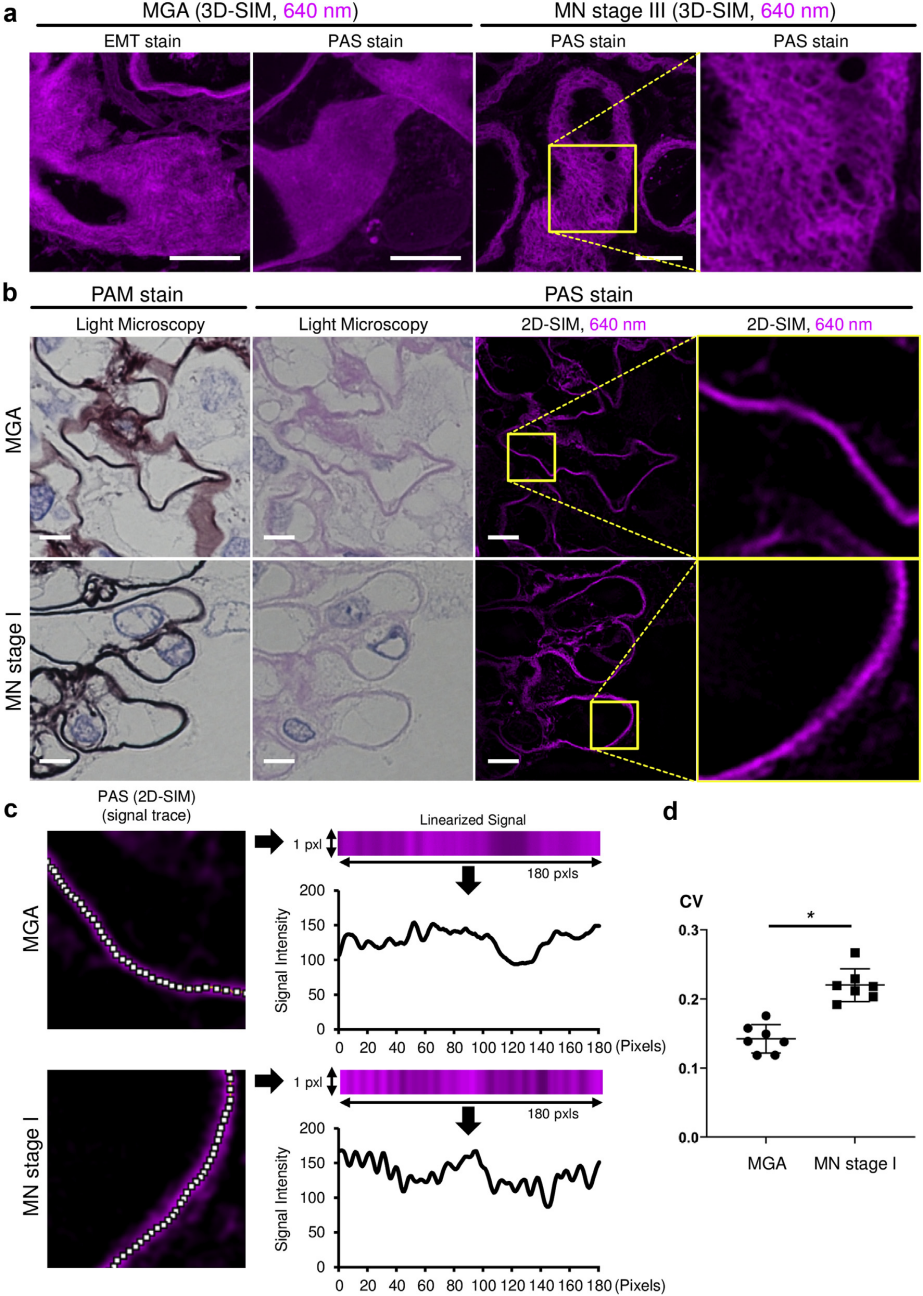
PAS staining in kidney sections can evaluate basement membrane integrity. (a) Representative three-dimensional structured illumination microscopy (3D SIM) images of elastica-Masson trichrome (EMT)– or periodic acid-Schiff (PAS)–stained glomeruli excited at 640 nm. Images were obtained from patients diagnosed with minor glomerular abnormalities (MGAs) (N = 7 patients) or membranous nephropathy (MN) stage III (N = 2 patients). (b) Representative light micrographs of periodic acid methenamine silver (PAM)– and PAS-stained specimens, and PAS-stained two-dimensional (2D) SIM images. Images were obtained from patients with MGA (N = 7 patients) or MN stage I (N = 7 patients). PAS-stained light microscopy and 2D-SIM images were obtained from the same kidney section portion (bars = 5 μm). (c) The analytical strategy for the basement membrane integrity. The regions of interest (1 × 180 pixels) were manually traced (white line in 2D SIM images) and then arranged into a straight line with ImageJ2 (linearized signal) for analysis. (d) Coefficient of variation (CV) was calculated based on the linearized signal intensities. PAS-stained human kidney sections from patients with MGA and MN stage I were analyzed. At least six images per patient were used for the analysis (N = 7 patients in each group, asterisk (∗) indicates significance at *P* = 0.0006, Mann-Whitney U test).

**Figure 3:**
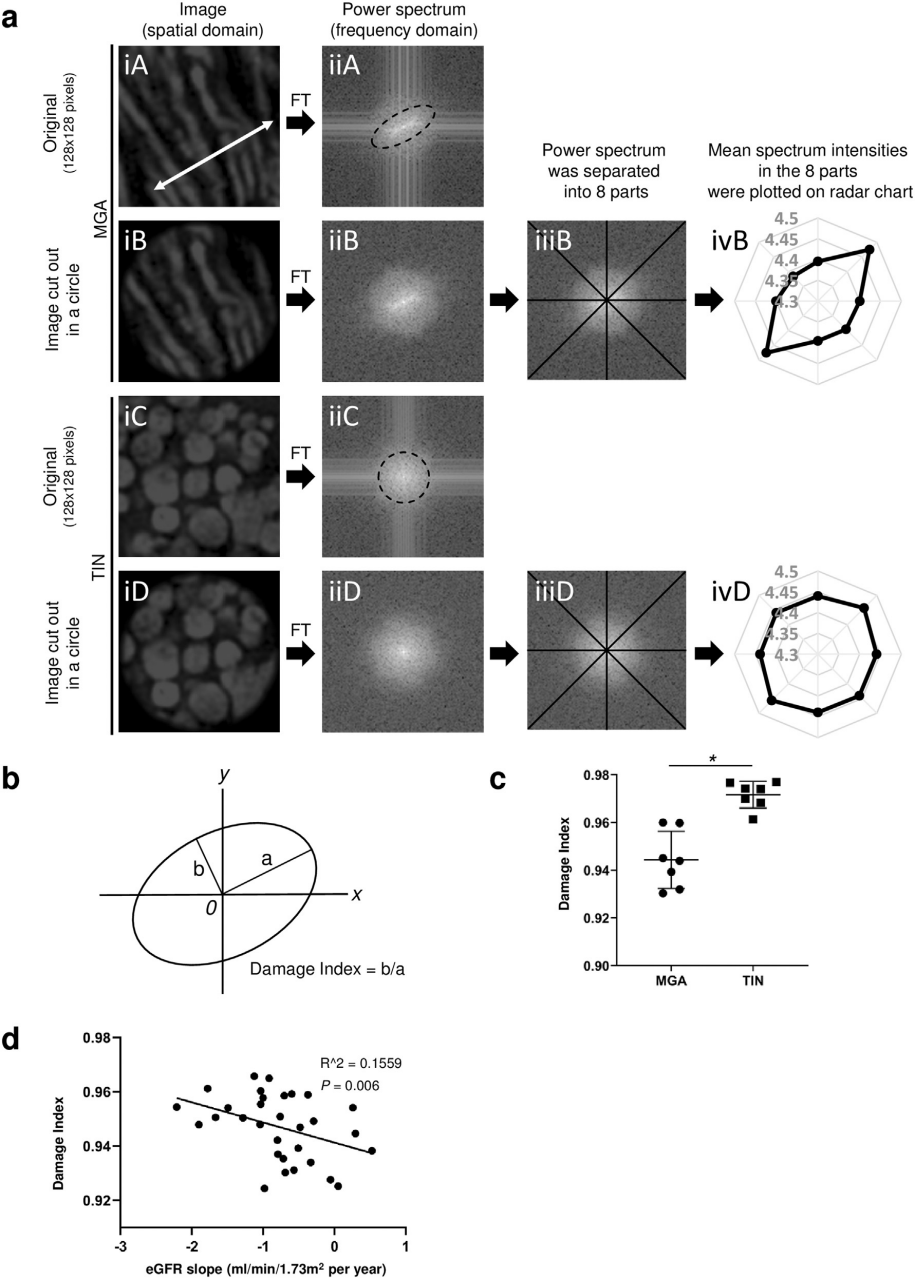
Quantitative analysis of mitochondrial changes in EMT-stained kidney specimens. (a) Quantifying strategy for mitochondrial damage is summarized. As shown in Figure 7a, mitochondrial two-dimensional structured illumination microscopy (2D SIM) images were obtained by exciting elastica-Masson trichrome (EMT)–stained kidney sections at 561 nm. The region of interest (ROI) (128 × 128 pixels) was manually cut out from the 2D SIM images. [a(iA)] shows a representative ROI cut out from 2D SIM images of seven patients with minor glomerular abnormalities (MGAs), whereas [a(iC)] shows a representative ROI from seven patients with tubulointerstitial nephritis (TIN). The white double-headed arrow in [a(iA)] indicates the direction of mitochondrial periodicity in the spatial domain. [a(iB)] and [a(iD)] images are circular cutouts from [a(iA)] and [a(iC)], respectively. Because Fourier transform (FT) of [a(iA)] and [a(iC)] generated cross-shaped noise in the frequency domain [a(iiA) and a(iiC)], the ROI in the spatial domain were cut out in circles. The dotted ellipse in [a(iiA)] represents the directionality in the frequency domain, which corresponds to the directionality shown as the white double-headed arrow in [a(iA)]. The dotted circle in [a(iiC)] indicates that mitochondrial images from patients with TIN did not yield directionality in the frequency domain. To quantify the directionality in frequency domain, power spectra were separated into eight parts [a(iiiB) and a(iiiD)], and then mean signal intensities in the eight parts were plotted on radar charts [a(ivB) and a(ivD)]. (b) An ellipsoidal approximation was applied to the radar chat. Schematic representation of the damage index for mitochondria, defined as the ratio of the minor axis to the major axis of the radar chart. (c) The mitochondrial damage indices were calculated based on 2D SIM images of specimens from patients with MGA and TIN. At least 21 images per patient were used for the calculations (N = 7 patients per group, ∗*P* < 0.05, Mann-Whitney U test). (d) Mitochondrial damage indices were measured in patients with immunoglobulin A nephropathy. The damage indices negatively correlated with estimated glomerular filtration rate slope after the kidney biopsy. At least 20 images per patient were used for the calculations (N = 31 patients, R^2^ = 0.1599; *P* = 0.006).

Kidney biopsy samples were obtained from inpatients of the Nephrology Unit at Osaka University Medical Hospital. Biopsy samples were fixed in a 10% neutral buffered formalin solution (Muto Pure Chemicals, Tokyo, Japan), dehydrated in an increasing ethanol concentration solution series, and embedded in paraffin. HE, PAS, or EMT staining was performed on 2-μm–thick kidney sections using standard procedures described in Tables S1–S4. For PAM staining, the extracellular matrix and cytoplasm are usually stained with eosin; however, the eosin staining process was routinely omitted at our facility. Samples were mounted in hydrophobic mounting medium. The staining procedures following the fixation were performed by SRL Co., Ltd. (Tokyo, Japan). Characteristics of the patients at the time of renal biopsy were obtained from their medical records and patients who were younger than 16 years of age were excluded from the study. For the analysis of the eGFR slope estimation, all patients diagnosed with IgA nephropathy from April 2009 to October 2010 in our faculty were included. The eGFR slope of each patient (ml/min/1.73 m^2^ per year) was estimated using a mixed-effects linear regression model including all available data on eGFR after kidney biopsy, with individual patients regarded as a random effect. The correlation between mitochondrial damage indices and clinical measurements was then analyzed using a robust linear regression model. All patients provided written informed consent and the ethics committee at the Osaka University Graduate School of Medicine approved this study (approval number 16375). Images for the foot process analyses (Figure 1c), coefficient of variation (CV) values (Figure 2b, c, and d), and damage indices (Figure 3c and d) were obtained by a physician who was blinded to patients’ information. The other images were not obtained in a blinded manner.

### Animals

Six-week–old male Sprague-Dawley rats and 8-week–old male C57BL/6 mice were purchased from Japan SLC Inc. (Shizuoka, Japan) and maintained at the animal facility of Osaka University School of Medicine.^26^^,^^27^ Puromycin aminonucleoside (PAN) nephrosis was induced in the rats with a single intravenous injection of PAN (Sigma-Aldrich, St. Louis, Missouri) dissolved in saline at a dose of 10 mg/100 g body weight. Kidney samples were obtained on day 7. For the lipopolysaccharide (LPS)–induced acute kidney injury model, mice were intraperitoneally injected with 10 mg/kg LPS (*Escherichia coli* O111:B4, Sigma-Aldrich). Kidney samples were obtained 18 hours after LPS injection.

For the ischemia reperfusion kidney injury model, bilateral renal pedicles were clamped for 30 minutes and kidney samples were obtained 15 minutes after reperfusion. Tissues were fixed with 4.0% paraformaldehyde in phosphate-buffered saline (pH 7.4), dehydrated, and embedded into paraffin.^28^ EMT staining was performed as described in Table S4 on 5-μm–thick kidney sections. Animals received humane and ethical treatment as outlined by the National Institutes of Health Guide for the Care and Use of Laboratory Animals.^29^ All animal experiments were approved by the Animal Committee of Osaka University (approval number 28-058-007).

### Bright Field Microscopy

Bright field microscopy images were obtained with a Nikon ECLIPSE E600 (Nikon, Tokyo, Japan) using an oil-immersion objective lens (100×).^30^

### Electron Microscopy

For electron micrographs, kidneys were dissected and fixed in 4.0% glutaraldehyde, post-fixed in 1.0% osmium tetroxide, and embedded in Epon-Araldite.^31^ Images were obtained with an H-7650 transmission electron microscope (Hitachi, Hitachi, Japan).

### SRM

Kidney sections stained with HE, PAS, PAM, and EMT were analyzed by N-SIM SRM (Nikon) with no additional florescent dye. To confirm that the structures visualized in the tubular area of the EMT-stained sections were mitochondria, we performed sequential staining for cytochrome c oxidase polypeptide IV (COX4, PM063, Medical & Biological Laboratories, Nagoya, Japan) before EMT staining.^32^, ^33^, ^34^ Briefly, COX4 immunofluorescent staining was performed on 2-μm paraffin-embedded sections and COX4 localization was visualized with SIM at 488-nm excitation. Then, the coverslip was removed, and the same sections were stained with EMT and excited with a 561-nm excitation laser.

### Emission Spectrum Analyses

Emission spectra of EMT staining dyes were measured with a SH-9000Lab plate reader (Corona, Ibaraki, Japan). Ponceau xylidine acid fuchsin azophloxine solution (#40251, Muto Pure Chemicals, Tokyo, Japan) and aniline blue (#40201, Muto Pure Chemicals) were diluted 50 times in distilled water and 8% acetic acid, respectively. The ranges of emission wavelengths for the spectra analyses were determined according to the emission filters equipped on the N-SIM. Spectra from the corresponding solvents were subtracted from the spectra of the ponceau xylidine acid fuchsin azophloxine solution, and aniline blue.

### Quantitative Analyses of Microscopic Images

To quantitatively analyze damage in podocyte foot processes and mitochondria, 128 × 128–pixel regions of interest (ROIs) corresponding to 4.13 × 4.13 μm were manually isolated from the original images. FT, inverse FT, and spectrum magnitude analyses were performed using NumPy 1.16.5, a library for scientific computing in Python. Circular cutouts of the mitochondrial images were also performed by NumPy in Python. Codes are available at the following website: https://github.com/NephrologyOsakaUniv/SuperResMicroscopy_2021. To analyze the integrity of the basement membrane, ROIs were traced in ImageJ2.^35^ At least 10 images per patient (N = 48 patients), 6 images per patient (N = 7 patients in each group), 21 images per patient (N = 7 patients in each group), and 20 images per patient (N = 31 patients) were analyzed in Figures 1c, 2d, and 3c and d, respectively.

### Statistical Analysis

Statistical analyses were performed with Stata/SE (version 16) software package (StataCorp, College Station, Texas) for eGFR slope estimation and analysis of association between mitochondrial damage indices and clinical measurements. GraphPad Prism8 (GraphPad Software, San Diego, California) was used for the others. Correlation between foot process integrity assessed by SIM and proteinuria was analyzed using simple linear regression. Comparisons between two groups were performed with the Mann–Whitney U test. Statistical analyses for variation of ratio were evaluated with chi-square test. Univariate and multivariate association between mitochondrial damage indices and clinical measurements were analyzed by robust linear regression analyses. Results were considered statistically significant at *P* < 0.05.

## Results

### Podocyte Foot Process Analysis in EMT-Stained Kidney Sections

**Figure 4:**
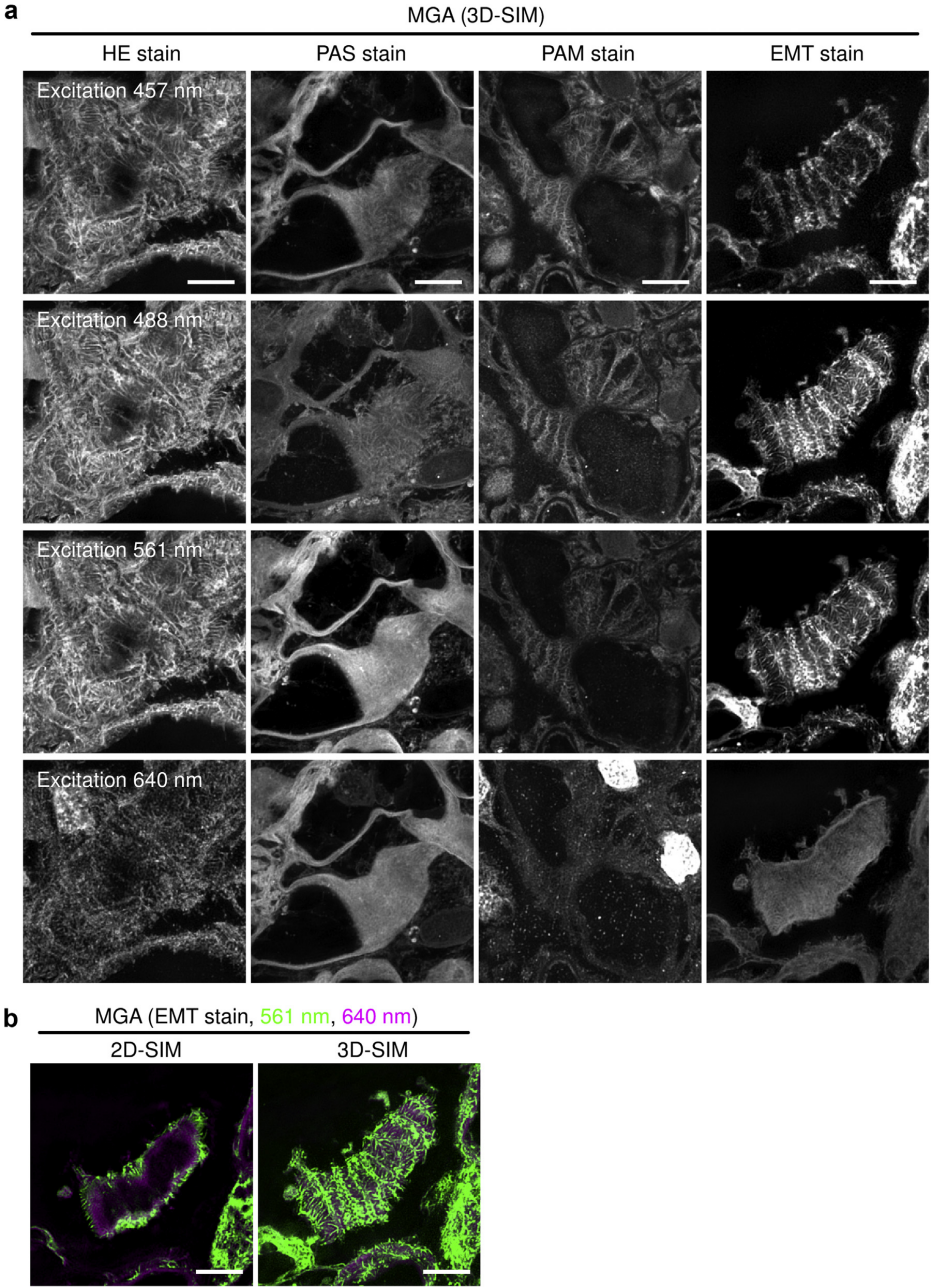
Podocyte foot process was visualized using elastica-Masson trichrome staining in kidney sections without any additional florescent probes. (a) Human kidney biopsy samples from patients diagnosed with minor glomerular abnormalities (MGAs) were analyzed. Paraffin-embedded 2-μm–thick kidney sections were stained with hematoxylin and eosin (HE), periodic acid-Schiff (PAS), periodic acid methenamine silver (PAM), or elastica-Masson trichrome (EMT). These sections were observed by three-dimensional structured illumination microscopy (3D SIM) with no additional fluorescent dye. Representative 3D SIM images of the glomeruli from seven MGA patients are shown. Several combination of staining methods and excitation lasers enabled to visualize podocyte foot processes and basement membrane (bars = 5 μm). Acquisition parameters are summarized in Table S5. (b) Representative podocyte foot process images obtained from EMT-stained sections using two-dimensional SIM (2D SIM) or 3D SIM. Microstructures were visualized with 561 nm and 640 nm excitation lasers and are pseudo-colored in green and magenta, respectively (bars = 5 μm).

Using SIM, we sought to visualize cellular microstructures of glomeruli in HE, PAS, PAM, or EMT-stained paraffin-embedded samples prepared as part of daily clinical practice. Staining protocols are summarized in Tables S1–S4. Human kidney sections from patients diagnosed with minor glomerular abnormalities (MGAs) were analyzed using 3D SIM. MGA is defined as an entity whose glomerular structure has minor changes using light microscopy, immunostaining, and electron microscopy, albeit with urinary abnormality.^36^ Scan parameters were optimized to obtain best signal/noise ratio (Table S5 and Figures S1–S4). The HE-stained sections excited at 457, 488, and 561 nm produced podocyte foot process images with a background signal from the glomerular basement membrane, whereas no obvious structures were visualized in the HE-stained sections excited at 640 nm (Figure 4a). PAS-stained sections visualized the glomerular basement membrane (Figure 4a). Foot processes were faintly visible in 488-nm–excited PAS-stained sections, although high-laser power and long exposure time were required (Figure 4a and Table S5). Additionally, PAM-stained sections excited at 457, 488, or 561 nm resulted in faint fluorescent signals of the foot process (Figure 4a). The PAM staining method used at our facility omits eosin-staining process (see Methods and Table S3); therefore, image acquisition of the PAM-stained sections required high laser power and long exposure times (Table S5). The EMT-stained sections were excited at 488 and 561 nm to visualize foot processes. In contrast to HE-stained sections, EMT-stained sections excited at 488 and 561 nm presented less signals from the background basement membrane, whereas sections excited at 457 nm presented a weak signal for the podocyte foot process (Figure 4a). Basement membranes could be visualized in EMT-stained sections with excitation at 640 nm (Figure 4a). Because EMT-stained sections, not HE-stained sections, enabled us to separately assess the foot process and the basement membrane, we used EMT-stained sections for further foot process analyses. Although excitation at both 488 and 561 nm detected the foot process in EMT-stained sections, we selected 561 nm because 1) phototoxicity is generally aggravated by shorter wavelengths and 1) image acquisition at 488 nm required a higher laser power and longer exposure time compared with acquisition at 561 nm (Table S5). In Figure 4a, we show images obtained with 3D SIM, whereas Figure 4b shows representative 2D and 3D SIM glomerular images obtained from EMT-stained sections at 561 and 640 nm excitation that were pseudo-colored in green and magenta, respectively. In comparison to 2D SIM, 3D SIM provided more comprehensive information about the structures of the foot process (Figure 4b).

**Figure 5:**
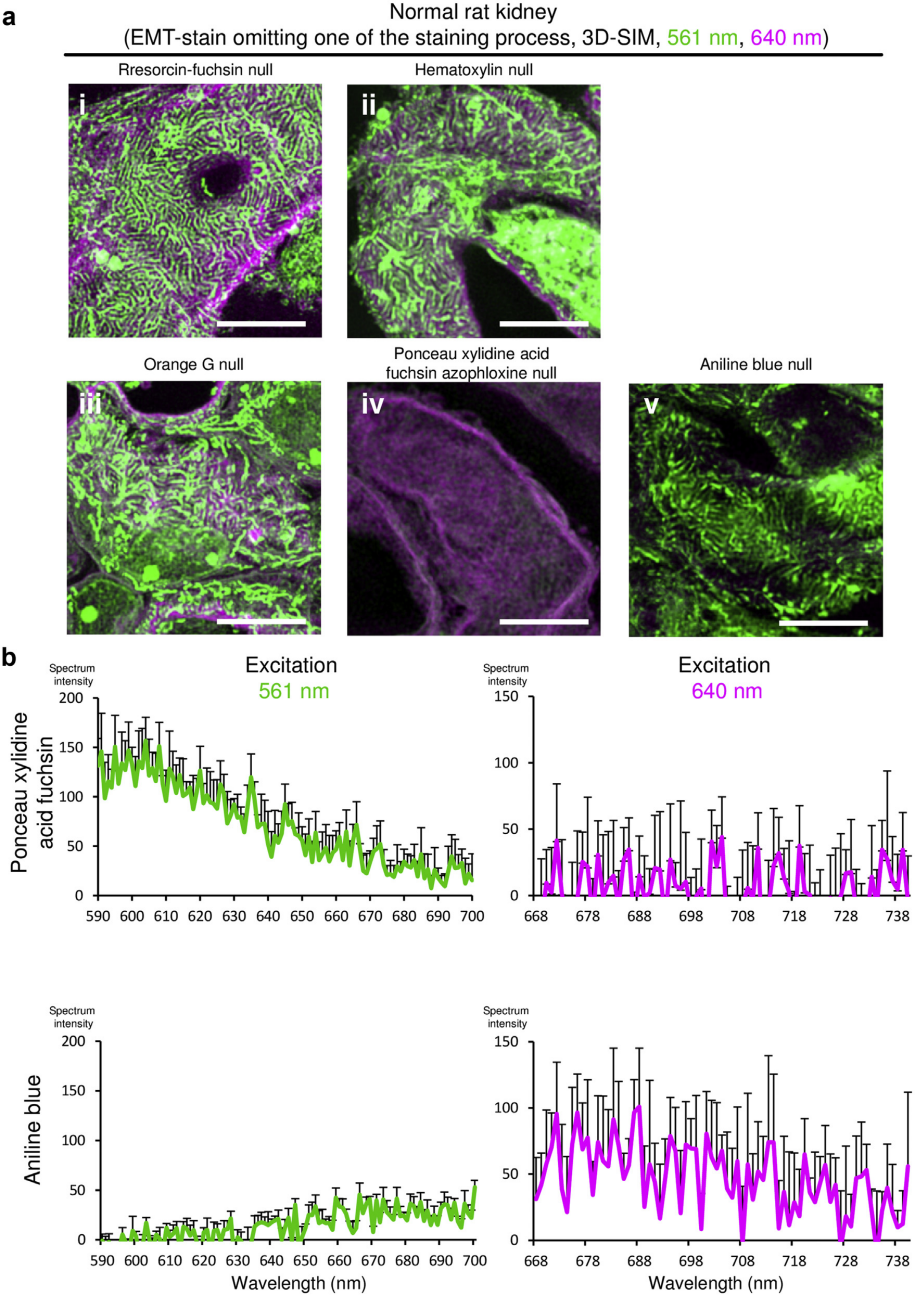
| Identification of fluorescent source in EMT-stained kidney samples. (a) Normal rat kidney tissues were stained using modified elastica-Masson trichrome (EMT) protocols that omitted (i) Maeda’s resorcin-fuchsin (procedure 3 in Table S4), (ii) Wiegert’s iron hematoxylin (procedure 8 in Table S4), (iii) orange G (procedure 13 in Table S4), (iv) ponceau xylidine acid fuchsin azophloxine (procedure 15 in Table S4), or (v) aniline blue (procedure 19 in Table S4). Representative three-dimensional structured illumination microscopy (3D SIM) images of the capillary walls from three animals in each staining protocol are shown (bars = 5 μm). Acquisition parameters are summarized in Table S5. Microstructures were visualized at 561-nm and 640-nm excitation and are pseudo-colored in green and magenta, respectively. (b) The fluorescence spectra of ponceau xylidine acid fuchsin azophloxine and aniline blue excited at 561- or 640-nm (N = 5 in each group). All results are presented as means ± standard deviation.

To analyze which dye in the EMT staining procedure (Table S4) caused fluorescence, normal rat kidney (Figure 5a) and human MGA (Figure S5) sections were stained with modified EMT protocols in which either resorcin-fuchsin, hematoxylin, orange G, ponceau xylidine acid fuchsin azophloxine, or aniline blue staining processes was omitted. The EMT staining without ponceau xylidine acid fuchsin azophloxine staining failed to visualize the foot process, whereas the basement membrane was nearly invisible in aniline-blue–null EMT staining (Figure 5a and S5). For the negative control analyses, we also observed unstained human and animal kidney sections under the same acquiring condition as EMT staining. Although weak background signals were detected in 457- and 488-nm–excited glomeruli, and in 457-, 488-, and 640-nm–excited tubules, no obvious structures were detectable (Figure S6 and S7). Fluorescence spectra confirmed that ponceau xylidine acid fuchsin azophloxine and aniline blue solutions fluoresced when excited at 561 and 640 nm, respectively (Figure 5b).

**Figure 6:**
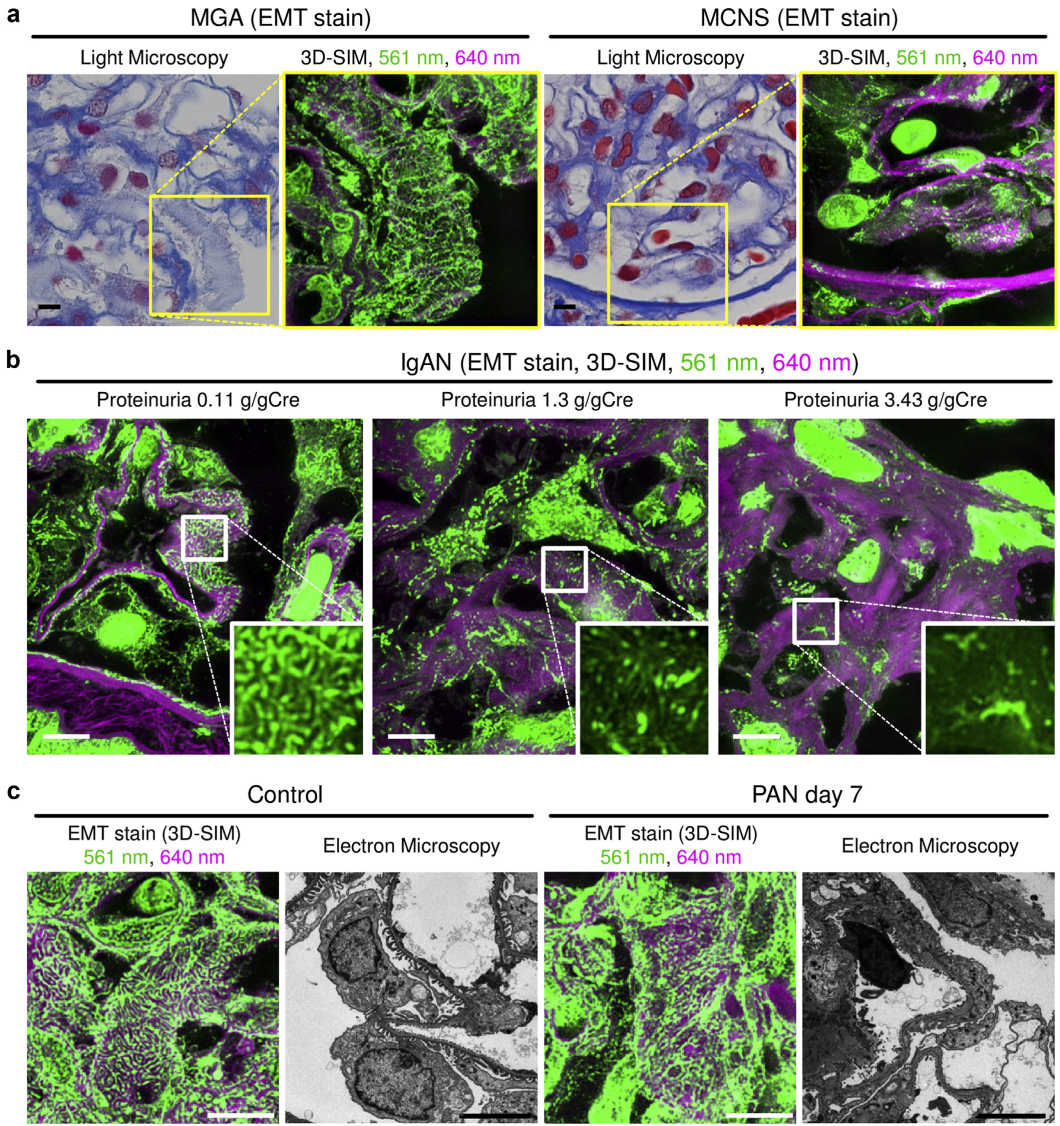
Three-dimensional SIM in combination with EMT-stained sections can visualize pathological changes of podocyte foot processes. (a) Kidney sections from patients diagnosed with minor glomerular abnormalities (MGAs) or minimal change nephrotic syndrome (MCNS) were stained with elastica-Masson trichrome (EMT) (bars = 2 μm). Microstructures visualized with 561-nm and 640-nm excitation lasers were pseudo-colored in green and magenta, respectively. Three-dimensional structured illumination microscopy (3D SIM), but not conventional light microscopy, can visualize the structural difference in podocyte foot process between patients with MGA and MCNS. Yellow squared regions in the light microscopic images were analyzed by SIM. (b) Kidney sections from patients with immunoglobulin A nephropathy (IgAN) with various levels of proteinuria were labeled with EMT staining and analyzed by 3D SIM (bars = 5 μm). White squares indicate areas analyzed under high magnification in the lower right of the panel. (c) Glomerulus from normal control rats and Puromycin aminonucleoside (PAN) nephrosis rats on day 7 were stained with EMT and analyzed by 3D SIM (bars = 5 μm). Transmission electron microscopy images of the same kidneys are also presented (bars = 2 μm) (N = 3 in each group).

We assessed whether EMT-based 3D SIM imaging could detect pathological changes in podocyte foot processes in two patients, one with MGA whose proteinuria was 0.49 g/gCr, and the other with minimal change nephrotic syndrome (MCNS) whose proteinuria was 13.2 g/gCr and whose foot process effacement was confirmed by electron microscopy (Figure S8a). Although there are certainly distinct differences in the structure of foot process between these two specimens, it was not detectable by conventional light microscopy; however, it was clearly visible using our method (Figure 6a). We further analyzed podocyte foot process structures in IgA nephropathy (IgAN) patients with various levels of proteinuria using EMT-based 3D SIM and observed foot processes in patients with massive proteinuria to be disrupted (Figure 6b). Moreover, disrupted foot process structure was also detected in a rat model of PAN nephropathy (Figure 6c). Hence, 3D SIM imaging using traditional EMT staining enabled us to evaluate podocyte foot processes.

### FT Can Quantify Foot Process Disruption

To quantify disrupted podocyte foot processes in kidney samples, we applied FT because normal foot processes have a periodical structure. FT is a mathematical transform that decomposes various signals, including images, into its constituent frequencies. We provide a schema that describes FT in Figure S9. Sine waves of different wavelengths were applied to 128 × 128–pixel squares to mimic the periodicity of the podocyte primary and foot processes (Figure 1a(i) and 1a(ii), respectively), and a representative ROI (128 × 128 pixels) from the foot process image was manually cut from the 3D SIM image of the 561-nm–excited EMT-stained sample (Figure 1a(iii)). Similar to the results shown in Figure S9, each original image in Figure 1a could be converted into PS by FT, whereas inverse FT reconverted the PS back into the original image. Moreover, inverse FT yielded images in a spatial domain when the PS were split into a concentric square and the remaining part of the PS (Figure 1a). The sine wave shown in Figure 1a(i), not the sine wave shown in Figure 1a(ii) or the foot process shown in 1a(iii), could be reconstructed from the PS, which lost the concentric square area. In contrast, the sine waves shown in Figure 1a(ii) and the foot process shown in 1a(iii), but not the sine wave in 1a(i), could be reconstructed from the concentric square area. Therefore, the information regarding the periodic structure of the foot process in the spatial domain was included within the concentric square region in the frequency domain (Figure 1a). We analyzed what portion of the PS best represented foot process integrity (Figure 1b). We split PS into various portions and found that a concentric square of (1/4)^2^-(1/16)^2^ adequately included information that represented normal foot process structures (Figure 1b). Using the PS intensities within a concentric square of (1/4)^2^-(1/16)^2^, we analyzed the integrity of the foot process in patients with MGA, MCNS, and IgAN (Figure 1c). There was no difference in age, sex, and serum creatinine levels among the patients in the MGA, MCNS and IgAN groups (Table 1). Levels of proteinuria were highest in the MCNS groups, whereas proteinuria of the IgAN group was significantly higher than that of the MGA group (Table 1). The PS intensities were negatively correlated with proteinuria (Figure 1c).

**Table 1:** Study population characteristics

Assay	Foot process (Figure 1c)			Basal membrane (Figure 2d)			Mitochondria (Figure 3d)
				MN	Mitochondria (Figure 3c)		
Group	MGA	MCNS	IgAN		MGA	TIN	IgAN
Number	4	6	38	7	7	7	31
Age, years	24 ± 9.41, ref	44.3 ± 16.0, ns	39.5 ± 14.7, ns	69 ± 5.18^a^	33.0 ± 14.6, ref	39.4 ± 21.0, ns	34.7 ± 14.1
Male (%)	50, ref	67, ns	53, ns	57, ns	29, ref	43, ns	45
Serum creatinine (mg/dl)	0.85 ± 0.17, ref	0.80 ± 0.18, ns	1.00 ± 0.57, ns	1.05 ± 0.53, ns	0.97 ± 0.20, ref	2.6 ± 2.79^b^	0.82 ± 0.20
Proteinuria (g/gCre)	0.17 ± 0.19, ref	7.11 ± 1.87^c^	1.00 ± 1.31^d^	8.00 ± 6.75^e^	0.41 ± 0.37, ref	0.29 ± 0.23, ns	0.57 ± 0.46

IgAN, immunoglobulin A nephropathy; MCNS, minimal change nephrotic syndrome; MGA, minor glomerular abnormality; MN, membranous nephropathy; ns, not significant; ref, reference; TIN, tubulointerstitial nephritis.

Summary of the patient characteristics shown in Figures 1c, 2d, 3c and 3d. Parameters at admission for renal biopsy are shown. All results are presented as mean values ± SD. Statistical significance for age, creatinine, and proteinuria among MGA, MCNS and IgAN for foot process was evaluated with analysis of variance followed by Dunnett’s *post hoc* test. Statistical significance for age, creatinine, and proteinuria between MGA and MN stage I, or between MGA and TIN for mitochondria was evaluated with Mann-Whitney U test. Statistical analysis for the gender ratio was evaluated with chi square test.

a *P* < 0.001.

b *P* < 0.05.

c *P* < 0.001.

d *P* < 0.01.

e *P* < 0.01.

### Basement Membrane Analysis in PAS-Stained Kidney Sections

Basement membranes could be visualized using PAS-stained sections (Figure 2a). Although 640-nm–excited EMT sections were also useful for visualizing the basement membrane, 640-nm–excited PAS-stained sections provided smoother and clearer images than 640-nm–excited EMT sections (Figure 2a). Therefore, we used PAS-stained sections to analyze glomerular basement membranes. As shown in Figure 2a, 3D SIM detected dimpled glomerular basement membranes in patients with membranous nephropathy (MN) stage III. Because pathological changes of glomerular basement membranes in MN stage III can be visualized not only by SIM but also by conventional light microscopy, we analyzed changes that are not detectable by conventional light microscopy, *i.e.,* glomerular basement membranes of MN stage I. Although electron microscopy has shown that there was subepithelial immunocomplex deposition in patients with MN stage I, no obvious difference between MGA and MN stage I was detectable using conventional light microscopy images (Figures S8b and 2b). We analyzed the same portion of PAS-stained sections using 2D SIM and found that the basement membrane in MN stage I patient samples had irregular signal intensity along the capillary wall line (Figure 2b). To quantify the irregularity, we traced the capillary wall and evaluated the CV values for the luminance (Figure 2c). Representative linearized signal intensity along with the capillary wall is shown in Figure 2c. Using these signal intensity values, we calculated the CV. Study population characteristics showed that subjects with MN stage I had severe proteinuria, whereas serum creatinine levels were not different between the MN and the MGA groups (Table 1). The CV values in patients with MN stage I were higher than those in patients with MGA, which reflected the irregularity of the fluorescent signal in these patients (Figure 2d).

### Mitochondrial Morphological Analysis in EMT-Stained Kidney Sections

We analyzed whether SIM can visualize microstructures in the tubular areas of the kidney. The HE, PAS, PAM, or EMT-stained paraffin-embedded kidney sections from patients diagnosed with MGA were analyzed by 2D SIM (Figure S10a) and among examined conditions; the EMT-stained sections excited at 561 nm clearly visualized mitochondria (Figure S10a). Acquisition parameters for mitochondrial imaging were summarized in Table S6. To confirm that the structures visualized in these EMT sections were mitochondria, we performed immunofluorescent staining of COX4 using a sample from patients with tubulointerstitial nephritis (TIN). Because EMT-stained sections were fluorescent under all SIM excitation lasers equipped in our facility (Figure S10a), we performed sequential staining of COX4 followed by EMT staining (Figure S10b; see Methods). In these sections, we observed structures excited by 561 nm in EMT sections that were surrounded by COX4 (Figure S10b).

**Figure 7:**
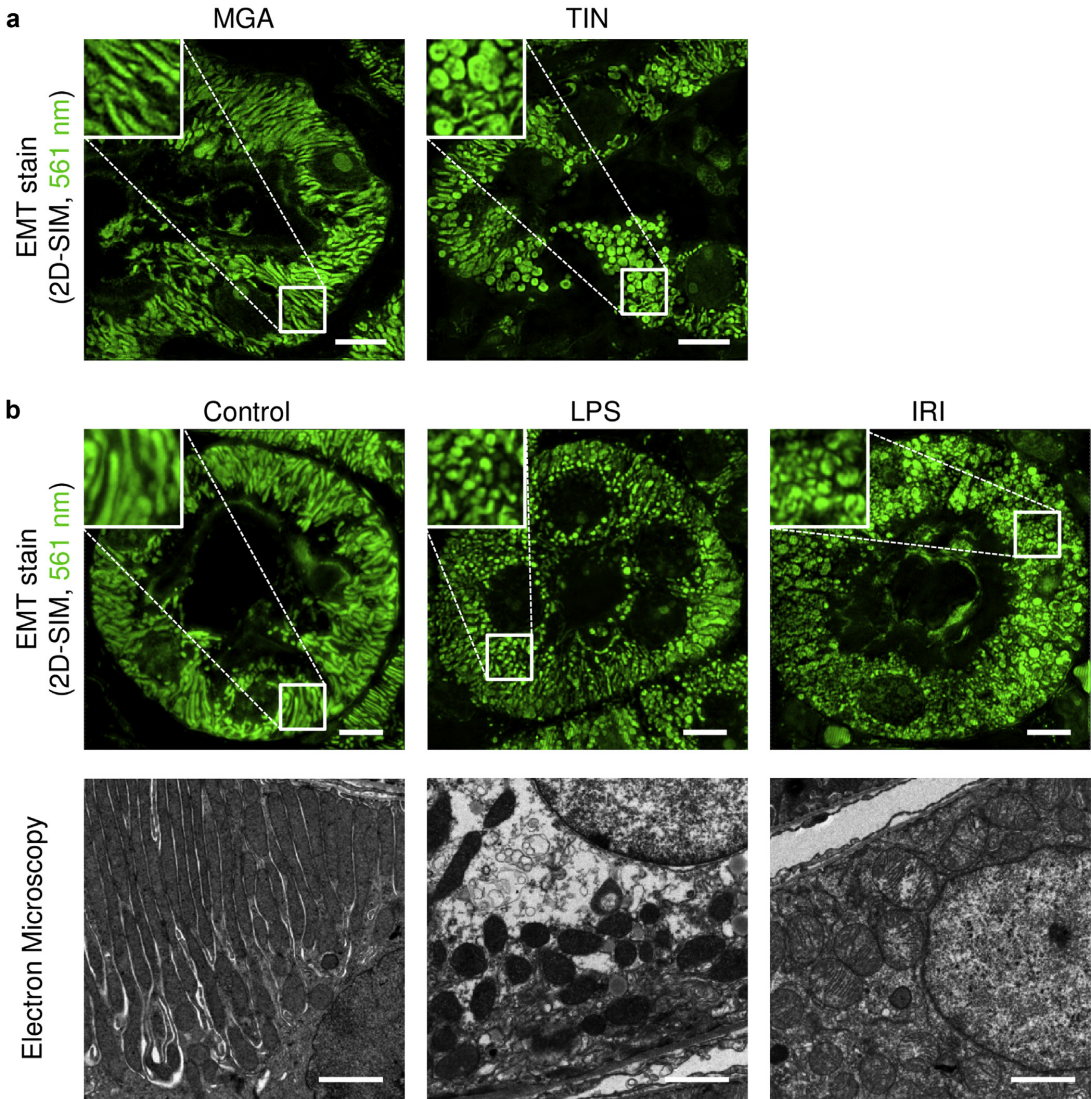
Mitochondria can be visualized with EMT-stained kidney sections. (a) Human kidney biopsy samples from patients diagnosed with minor glomerular abnormalities (MGAs) or tubulointerstitial nephritis (TIN) were stained with elastica-Masson trichrome (EMT). Two-dimensional structured illumination microscopy (2D SIM) images excited at 561 nm were pseudo-colored in green. Acquisition parameters are summarized in Table S6. Fragmented mitochondria were detected in the kidney sections from patients with TIN (N = 7 patients per group). White squares indicate areas analyzed under high magnification in the upper left of the panel. (b) Changes in mitochondrial morphology induced by lipopolysaccharide (LPS) or ischemia reperfusion injury (IRI) were analyzed in EMT-stained murine kidney sections with 2D SIM (bars = 5 μm). Electron microscopy images of the corresponding kidney tissues are also shown to illustrate that LPS induced mitochondrial fragmentation and that IRI caused mitochondrial swelling (bars = 2 μm; N = 3 to 4 per group).

We then analyzed whether EMT-based 2D SIM imaging could detect pathological changes in mitochondria. Because severe stress facilitates mitochondrial fission and thereby accelerates removal of injured mitochondria, changes in mitochondrial morphology can reflect mitochondrial damages. In contrast to long tube-like structures observed in the kidney sections from patients with MGA, fragmented mitochondria were detected in the sections from patients with TIN (Figure 7a). Considering that LPS and ischemia reperfusion kidney injury have been reported to promote mitochondrial fission in mice, we also analyzed whether EMT-based 2D SIM could detect mitochondrial changes in these models.^37^^,^^38^ We clearly observed mitochondrial fragmentation and swelling which were confirmed by electron microscopy when these animal models were assayed using our SIM imaging method (Figure 7b).

### FT Can Quantify Morphological Changes in Mitochondria

To quantify the mitochondrial changes observed by SIM, we applied FT to the micrographs. ROIs (128 x 128 pixels) of patients with MGA and TIN were manually cut out from 561-nm–excited EMT-stained section images [Figure 3a(iA) and a(iC)]. Because the structure of normal mitochondria in the spatial domain had periodicity in one direction [white double-headed arrow in Figure 3a(iA)], a high signal intensity was observed in the same direction in the frequency domain [dotted ellipse in Figure 3a(iiA)]. In contrast, mitochondrial images from patients with TIN did not present a directional signal in the frequency domain [Figure 3a(iiC)]. Considering that FT of 128 × 128 pixels ROIs generated cross-shaped noise in the frequency domain, the 128 × 128 square images in the spatial domain were cut into circles [Figure 3a(iB) and a(iD)] that did not generate cross-shaped noise in the frequency domain [Figure 3a(iiB) and a(iiD)]. To quantify the directionality in the frequency domain, the PS were separated into eight parts, and mean signal intensity in these parts were plotted on radar charts [Figure 3a(iiiB), a(ivB), a(iiiD), and a(ivD)]. As shown in Figure 3a, normal mitochondrial image yielded a radar chart of ellipse-like shape, whereas injured mitochondrial image yielded a radar chart of circular shape. These findings indicated that mitochondrial damages can be evaluated by calculating the ratio of the minor to major axes in the radar charts (damage index in Figure 3b). We measured the major and minor axes of the radar charts and calculated the damage index, defined as the ratio of the minor axis to the major axis (Figure 3b). We evaluated the damage indices in patients with MGA or TIN whose characteristics indicated that the kidneys of the TIN patients were severely injured (Table 1). The damage indices of the TIN patients were significantly higher than those of MGA patients (Figure 3c). We further analyzed the damage indices in IgAN patients who underwent kidney biopsies from April 2009 to October 2010 (Table 1). The damage index negatively correlated with eGFR slope in robust linear regression model, indicating that the damage index predicted future decline in kidney function (Figure 3d and Table 2). Furthermore, in a multivariate robust linear regression model, the damage index was significantly associated with the eGFR slope even after adjustment for other predictors for the progression of IgAN baseline eGFR, proteinuria, and T-score of Oxford Mesangial hypercellularity, Endocapillary hypercellularity, Segmental glomerulosclerosis, Tubular atrophy/interstitial fibross, Cellular or fibrocellular crescents (MEST-C) (Table 2). There were no significant associations between the damage index and baseline eGFR (Figure S11a), proteinuria (Figure S11b), and T-score of Oxford MEST-C (Figure S11c). These results show that 2D SIM imaging using traditional EMT staining is useful to evaluate mitochondrial damages in the tubules.

**Table 2:** Robust linear regression analysis for an association between the eGFR slope and mitochondrial damage index

	Univariate			Multivariate^a^		
	β	*P*	95% CI	β	*P*	95% CI
Damage index, per 0.1 unit increase	-2.10	0.006	-3.56 to -0.65	-1.60	0.03	-3.02 to -0.18

β, β-coefficient; CI, confidence interval; eGFR, estimated glomerular filtration rate; MEST-C, Mesangial hypercellularity, Endocapillary hypercellularity, Segmental glomerulosclerosis, Tubular atrophy/interstitial fibross, Cellular or fibrocellular crescents.

The unit of eGFR slope was ml/min/1.73 m^2^ per year.

a Model adjusted for proteinuria, T-score of MEST-C, and eGFR at biopsy.

## Discussion

Herein, we have shown that EMT-stained kidney sections are useful for quantifying the integrity of podocyte foot processes and tubular mitochondria, whereas PAS-stained sections are appropriate for analysis of basement membrane microstructures. Because our method does not require additional sample preparation, we obtained the microstructural information from human specimens without loss of samples. Moreover, we developed a novel FT-based quantitative method to evaluate the integrity of foot processes and mitochondria, the morphological changes of which directly cause kidney diseases. Subtle changes in the basement membrane in MN stage I were quantified by calculating CV values. Hence, microstructural information of the kidney was obtained from sections prepared as part of a routine clinical practice for a renal disease patient.

Electron microscopy is the gold standard method for detailed analysis of cellular microstructures. Although SRM can surpass the diffraction limit of visible light, electron microscopy can achieve much higher resolution because the electron beam wavelength is much shorter than visible light.^39^^,^^40^ Therefore, SRM cannot replace electron microscopy in terms of resolution, making the latter necessary for analyses of cellular microstructures. However, SRM can offer several advantages over electron microscopy. First, SRM analysis can be applied to paraffin-embedded sections rather than epon-embedded sections, allowing for the visualization of cellular microstructures in patient samples that are not processed as epon-embedded blocks at the time of renal biopsy. Second, SRM can survey a wider range of specimens, which is critical for quantitative analyses of histological changes. Third, SRM can reveal 3D structures. Because of this, our strategy enabled us to evaluate the detailed architecture of foot processes. Although scanning electron microscopy can visualize superficial 3D structures, the electron beam surveys only the surface of a specimen. Because excitation lasers can penetrate sections, SRM can visualize both the surface and inner microstructures of the specimen, enabling visualization of basement membranes beneath podocytes. These points indicate that electron microscopy and SRM can play complementary roles in better understanding kidney disease.

Several methods have been applied to quantify injured foot processes. For example, Deegens *et al.*^2^ showed that foot process width could distinguish idiopathic focal segmental glomerulosclerosis from secondary focal segmental glomerulosclerosis. Ichimura *et al.*^41^ measured the length of the foot process using focused-ion beam/scanning electron microscopy to analyze morphological changes during the progression of PAN nephrosis. Unnersjö-Jess *et al.*^19^ quantified the length of the podocin-stained slit diaphragm from stimulated emission depletion microscopy images to quantify foot process effacement in passive Heyman nephritis.^13^ More recently, Butt *et al.*^13^ quantified the length, width, area, perimeter of foot process, slit diaphragm length, and slit diaphragm grid index from nephrin-stained stimulated emission depletion microscopy images, and thereby demonstrated that morphological alterations of the glomerular filtration barrier lead to albuminuria. Although foot processes have a sophisticated 3D structure, most of these previously reported quantification methods used only 1D information. Moreover, they require time-consuming tracing of the foot process from these images. In contrast, our FT-based method uses 2D information and does not require tracing, thus strengthening the application of our FT-based quantification method.

### Study Limitations

There were several limitations associated with our study. First, SIM is not currently available or present in many general hospitals. Therefore, collaboration between hospitals and research facilities that have SIM is required for the broad application of our strategy. Second, we analyzed only relatively thin sections (2-μm–thick sections in human renal biopsy and 5-μm–thick sections in animal samples) because our facility routinely uses 2 μm-thick sections for the preparation of kidney biopsy samples. As many conventional histology stains can result in uneven staining in thick specimens, fluorescent labeling of abundant reactive entities, which can covalently stain thick sections, may be helpful in the analyses of thick samples.^42^ Third, this study is a single center study, thus we have not evaluated how inter-laboratory differences in histological staining protocols affect the performance of SIM in visualizing kidney microstructures. However, human and animal samples in this study were stained by different facilities. Human sample staining was carried out by SRL Co., Ltd., while animal samples were stained in our facility. Therefore, inter-laboratory variability in histological staining may not severely affect the performance of SIM, yet a multicenter study is required to precisely evaluate effects of inter-laboratory variability. Fourth, image acquisition speeds of N-SIM, the super-resolution microscopy used in this study, were about 1 and 5 min per image for 2D-SIM and 3D-SIM, respectively. N-SIM can provide an image of 32.7725 x 32.7725 μm size at once. Therefore, it takes several tens of minutes to evaluate entire glomeruli and multiple tubules. However, acquisition speed of N-SIM S, a newer version of N-SIM that our facility is not yet equipped with, is about ten times faster than N-SIM. Therefore, SIM can be used to evaluate entire glomeruli and multiple tubules in a reasonable amount of time, at least with N-SIM S. Fifth, in contrast to the established usefulness of electron microscopy in the kidney biopsy evaluation, usability of SIM as a clinical diagnostic tool is still in its infancy. Further studies are required to establish the usefulness of SIM in clinical settings.

In conclusion, we developed a novel strategy to facilitate the quantification of microstructural changes in kidney biopsy specimens. PAS- or EMT-stained sections prepared during daily clinical practice are sufficient to extract information regarding podocyte foot processes, mitochondria, and basement membranes. Meanwhile, electron microscopy and SRM can play complementary roles in better understanding kidney disease pathology.

## Disclosures

Supported by a grant from 10.13039/501100001691JSPS KAKENHI 18K15974.

## Supplementary Material

Supplementary File (PDF)

**Table S1.** Hematoxylin-eosin staining procedure.

**Table S2.** Periodic acid-Schiff staining procedure.

**Table S3.** Periodic acid methenamine silver staining procedure.

**Table S4.** Elastica Masson trichrome staining procedure.

**Table S5.** Acquisition parameters for 3D-SIM foot process imaging.

**Table S6.** Acquisition parameters for the 2D-SIM mitochondrial imaging.

**Figure S1.** Optimization of scan parameters for HE stained sections.

**Figure S2.** Optimization of scan parameters for PAS stained sections.

**Figure S3.** Optimization of scan parameters for PAM stained sections.

**Figure S4.** Optimization of scan parameters for EMT stained sections.

**Figure S5.** Human MGA kidney tissues were stained using modified EMT protocols.

**Figure S6.** Micrographs obtained from unstained human kidney specimens.

**Figure S7.** Micrographs obtained from unstained animal kidney specimens.

**Figure S8.** Electron micrographs of patient specimens shown in Figures 2a and 6b.

**Figure S9.** A concise illustration of Fourier transform.

**Figure S10.** Mitochondrial structures were visualized in elastic-Masson trichrome–stained kidney sections.

**Figure S11.** Mitochondrial damage index and clinical measurements.
