# Supplementary material for: Quantitative Analyses of Foot Processes, Mitochondria, and Basement Membranes by Structured Illumination Microscopy Using Elastica-Masson– and Periodic-Acid-Schiff–Stained Kidney Sections
Source: Kidney Int Rep. 2021 May 1;6(7):1923–38. doi: 10.1016/j.ekir.2021.04.021 (PMC8258503; doi:10.1016/j.ekir.2021.04.021)
Supplement: Supplementary File (PDF) [file mmc1.pdf]

Supplemental Material Table of Contents

Table S1. Hematoxylin-eosin staining procedure

Table S2. Periodic acid-Schiff staining procedure

Table S3. Periodic acid methenamine silver staining procedure

Table S4. Elastica Masson trichrome staining procedure

Table S5. Acquisition parameters for 3D-SIM foot process imaging

Table S6. Acquisition parameters for the 2D-SIM mitochondrial imaging

Figure S1. Optimization of scan parameters for HE stained sections

Figure S2. Optimization of scan parameters for PAS stained sections

Figure S3. Optimization of scan parameters for PAM stained sections

Figure S4. Optimization of scan parameters for EMT stained sections

Figure S5. Human MGA kidney tissues were stained using modified EMT protocols

Figure S6. Micrographs obtained from unstained human kidney specimens

Figure S7. Micrographs obtained from unstained animal kidney specimens

Figure S8. Electron micrographs of patient specimens shown in Figures 6a and 2b

Figure S9. A concise illustration of Fourier transform

Figure S10. Mitochondrial structures were visualized in elastica Masson trichrome-

stained kidney sections

Figure S11. Mitochondrial damage index and clinical measurements

Supplementary information is available at *KI reports's* website.

Table S1. Hematoxylin-eosin staining procedure

Procedure	Time
1. Deparaffinize and hydrate to deionized water	
2. Mayer's hematoxylin (Muto Pure Chemicals #30002)	10 min
3. Wash in running tap water	1 min
4. Differentiation in distilled water	15 min
5. New Eosin Type M solution (Muto Pure Chemicals #32081)	10 min
6. Dehydrate in 75% ethanol	1 min
7. Dehydrate in 90% ethanol	1 min
8. Dehydrate in 100% ethanol	3 min, 3 times
9. Clear and mount	

Table S2. Periodic acid-Schiff staining procedure

Procedure	Time
1. Deparaffinize and hydrate to deionized water	
2. 1% Periodic acid solution (Muto Pure Chemicals #40911)	10 min
3. Wash in running tap water	5 min
4. Rince in distilled water	2 min
5. Cold Schiff's reagent (Muto Pure Chemicals #40931)	3 min
6. Sodium bisulfate solution (Muto Pure Chemicals #40941)	2 min, 3 times
7. Wash in running tap water	5 min
8. Mayer's hematoxylin (Muto Pure Chemicals #30002)	2 min
9. Wash in running tap water	1 min
10. Differentiation in distilled water	10 min
11. Dehydrate in 75% ethanol	1 min
12. Dehydrate in 90% ethanol	1 min
13. 100% dehydrate in ethanol	3 min, 3 times
14. Clear and mount	

Table S3. Periodic acid methenamine silver staining procedure

Procedure	Time
1. Deparaffinize and hydrate to deionized water	
2. 1% Periodic acid solution (Muto Pure Chemicals #40911)	10 min
3. Wash in running tap water	5 min
4. Rince in distilled water	2 min
5. 0.5% Thiosemicarbazide solution (Wako #204-01182)	5 min
6. Wash in running tap water	5 min
7. Rince in distilled water	2 min
8. Methenamine silver solution at 60℃ (0.75% hexamethylenetetramine (Wako, #081-00332), 0.125% silver nitrate (Wako, #194-00832), 0.125% sodium borate (Kanto chemical, #37127-00))	15 min
9. Rince in distilled water	2 min
10. 0.2% gold (Ⅲ) chloride (Wako, #086-05321)	5 min
11. Wash in running tap water	5 min
12. Rince in distilled water	2 min
13. 2% sodium thiosulfate (Wako, #197-03605)	5 min
14. Wash in running tap water	5 min
15. Rince in distilled water	2 min
16. Mayer’s hematoxylin (Muto Pure Chemicals #30002)	2 min
17. Wash in running tap water	1 min
18. Differentiation in distilled water	10 min
19. Dehydrate in 75% ethanol	1 min
20. Dehydrate in 90% ethanol	1 min
21. 100% dehydrate in ethanol	3 min, 3 times
22. Clear and mount	

Table S4. Elastica Masson trichrome staining procedure

Procedure	Time
1. Deparaffinize and hydrate to deionized water	
2. 70% ethanol	quickly
3. Maeda's resorcin-fuchsin solution (Muto Pure Chemicals #40321)	15 min
4. Rinse in 100% ethanol	quickly
5. Wash in running tap water	3 min
6. First mordant (Muto Pure Chemicals #40061) (5% trichloroacetic acid, 5% potassium dichromate	20 min
7. Wash in running tap water	3 min
8. Weigert's iron hematoxylin (Muto Pure Chemicals #40341, 40351)	10 min
9. Wash in running tap water	10 min
10. Second mordant (Muto Pure Chemicals #81411) (2.5% phosphomolybdic acid, 2.5 % phosphotungstic acid)	30 s
11. Wash in running tap water	1 min
12. 1% acetic acid	quickly
13. 0.75% Orange G (Muto Pure Chemicals #40231)	1 min
14. 1% acetic acid	quickly
15. Ponceau xylidine acid fuchsin azophloxine solution (Muto Pure Chemicals #40251)	15 min
16. 1% acetic acid	quickly
17. 2.5% phosphotungstic acid (Muto Pure Chemicals #40181)	15 min
18. 1% acetic acid	quickly
19. Aniline blue (Muto Pure Chemicals #40201)	10 min
20. 1% acetic acid	quickly
21. Dehydrate in 100% ethanol	3 min, 3 times
22. Clear and mount	

Table S5. Acquisition parameters for 3D-SIM foot process imaging

Parameter	HE	PAS	PAM	EMT
Laser power (%)				
457 nm	20	40	80	60
488 nm	10	80	80	90
561 nm	50	30	90	50
640 nm	90	40	80	30
Exposure time (ms)				
457 nm	20	100	600	200
488 nm	50	800	400	600
561 nm	100	100	800	200
640 nm	800	200-800	600	100
Gain multiplier	250	250	300	250
Conversion gain	1×	1×	1×	1×

Abbreviations: 3D-SIM, 3-dimensional structured illumination microscopy; HE, hematoxylin and eosin; PAS, periodic acid Schiff; PAM, periodic acid methenamine silver; EMT, elastica Masson trichrome.

Table S6. Acquisition parameters for the 2D-SIM mitochondrial imaging

Parameters	HE	PAS	PAM	EMT
Laser power (%)				
457 nm	5	40	80	20
488 nm	8	80	80	90
561 nm	15	50	90	20
640 nm	80	90	80	40
Exposure time (ms)				
457 nm	30	100	200	100
488 nm	30	800	600	600
561 nm	70	70	800	100
640 nm	600	800	600	200
Gain multiplier	250	250	300	250
Conversion gain	1×	1×	1×	1×

Abbreviations: 2D-SIM, 2-dimensional structured illumination microscopy; HE, hematoxylin and eosin; PAS, periodic acid Schiff; PAM, periodic acid methenamine silver; EMT, elastica Masson trichrome.

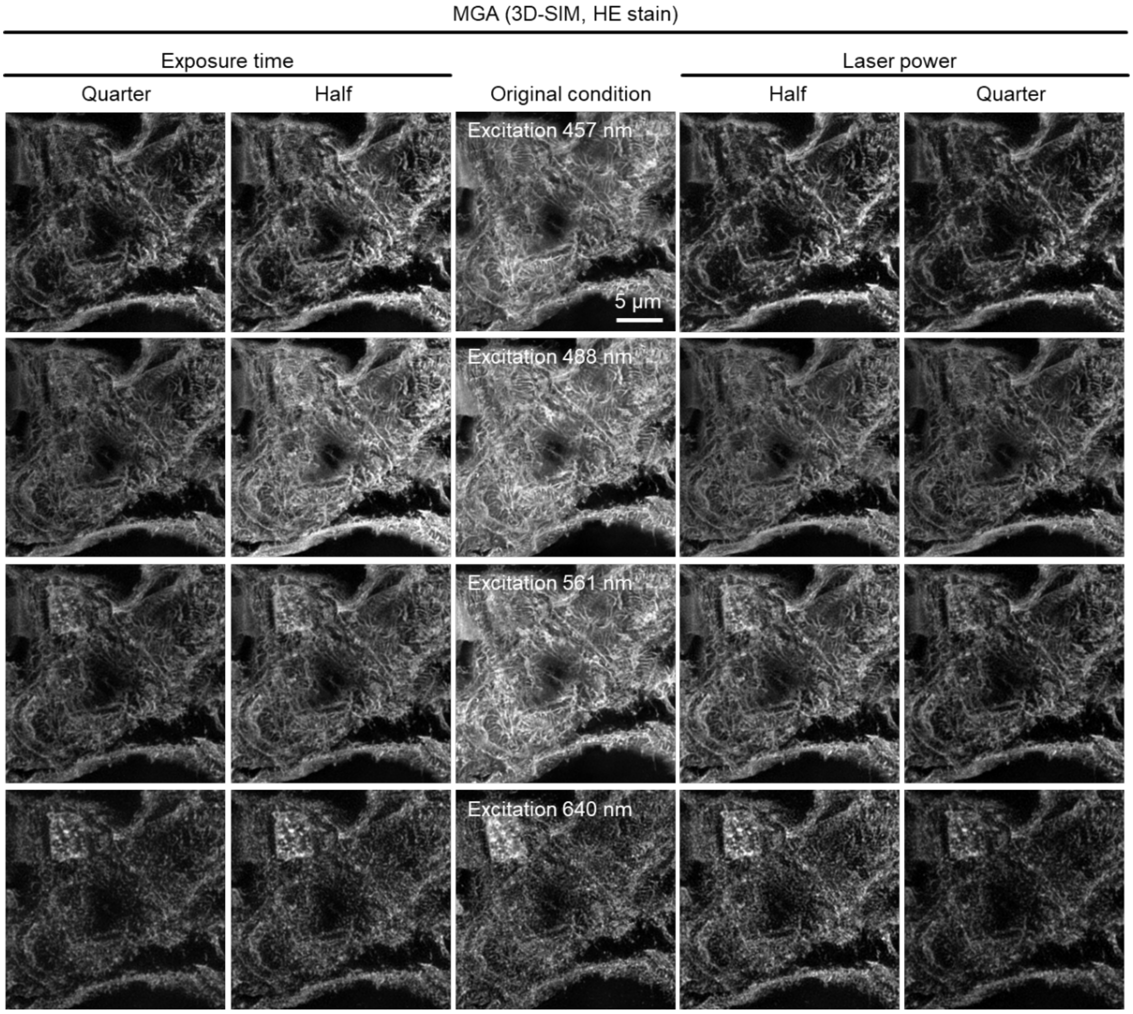

Figure S1. Optimization of scan parameters for HE stained sections

Scan parameters of 3D-SIM were optimized to obtain best signal/noise ratio. Human kidney biopsy samples from patients diagnosed with minor glomerular abnormalities (MGA) were analyzed. Original condition indicates scan parameters shown in Table S5. Images obtained with various exposure times and laser powers are shown.

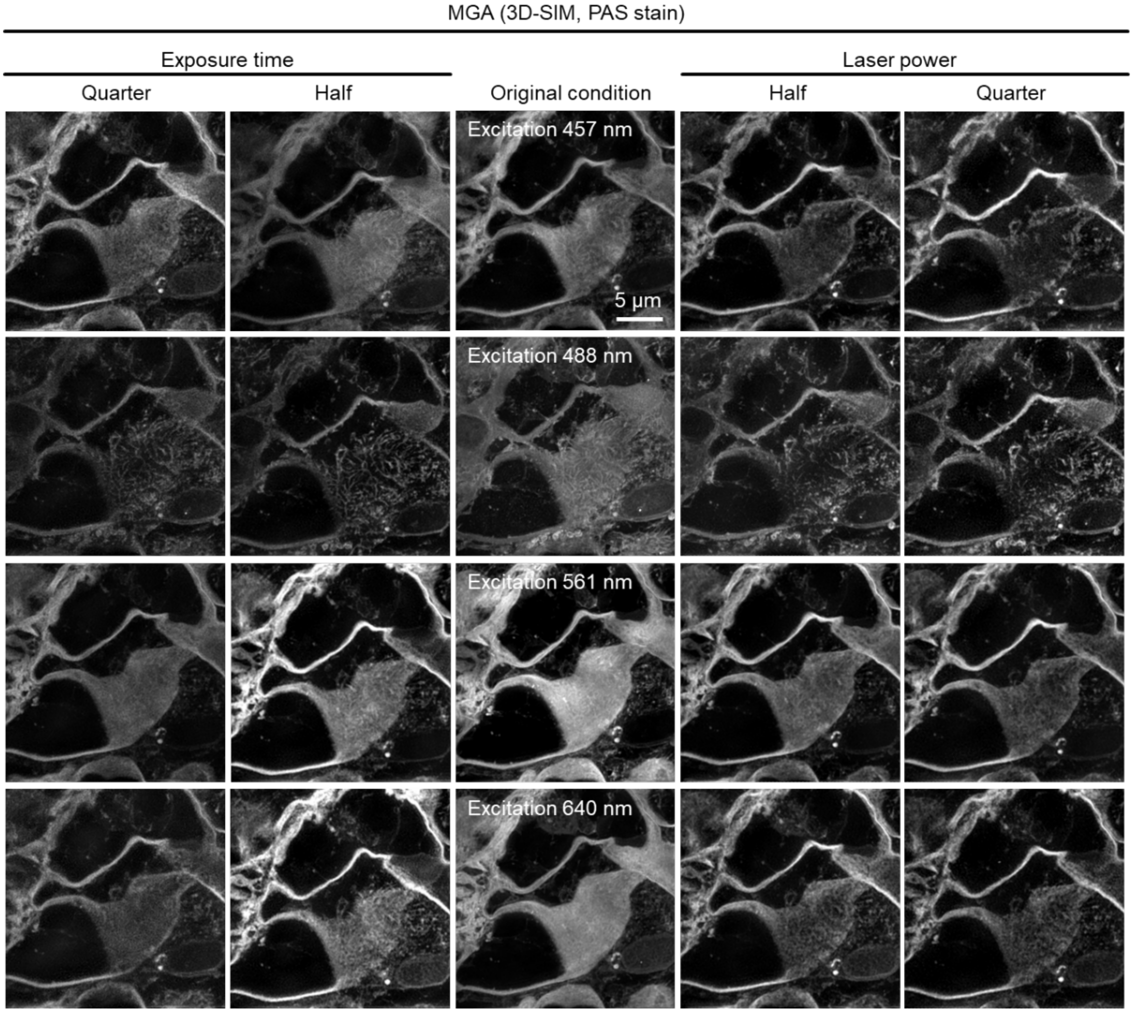

Figure S2. Optimization of scan parameters for PAS stained sections

Scan parameters of 3D-SIM were optimized to obtain best signal/noise ratio. Human kidney biopsy samples from patients diagnosed with minor glomerular abnormalities (MGA) were analyzed. Original condition indicates scan parameters shown in Table S5. Images obtained with various exposure times and laser powers are shown.

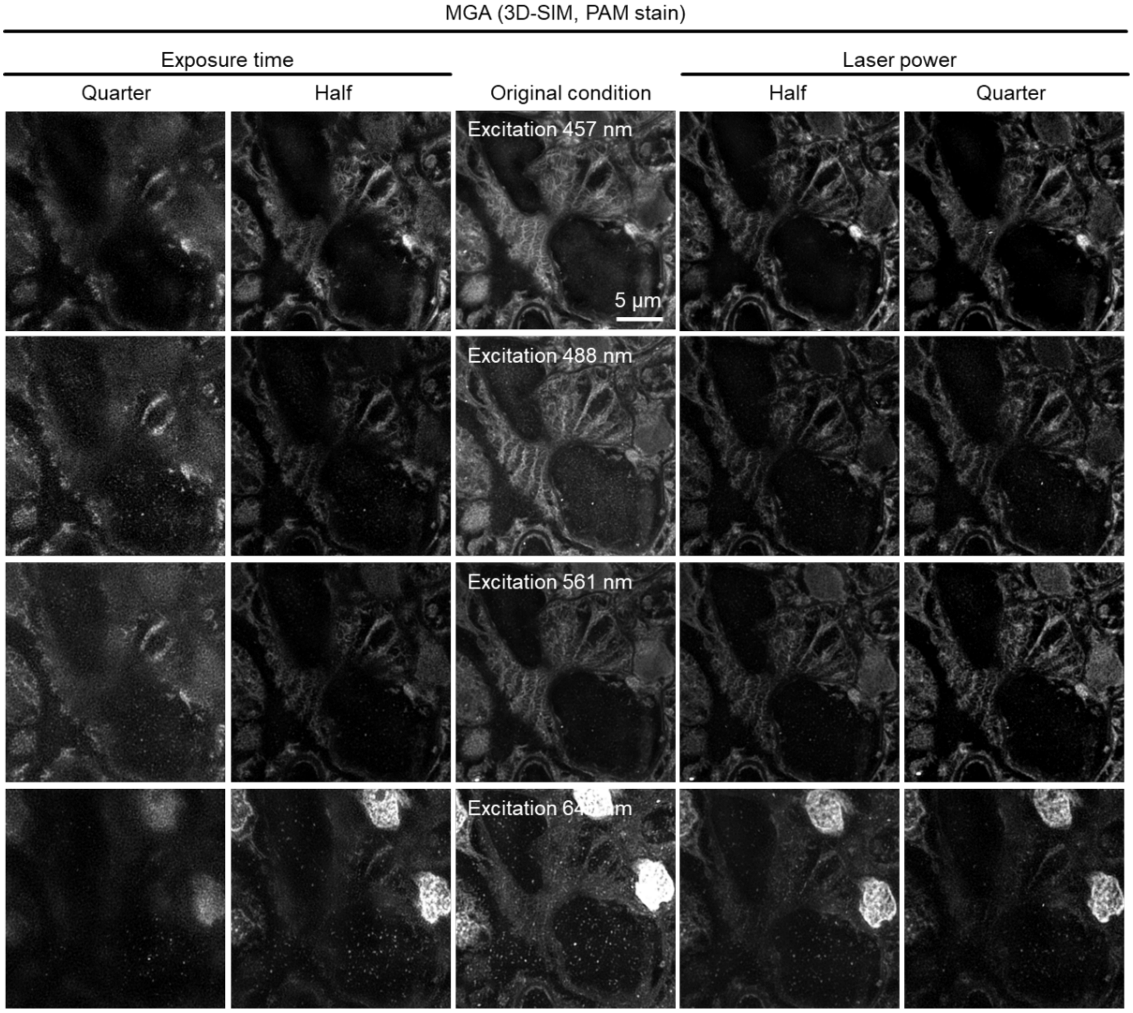

Figure S3. Optimization of scan parameters for PAM stained sections

Scan parameters of 3D-SIM were optimized to obtain best signal/noise ratio. Human kidney biopsy samples from patients diagnosed with minor glomerular abnormalities (MGA) were analyzed. Original condition indicates scan parameters shown in Table S5. Images obtained with various exposure times and laser powers are shown.

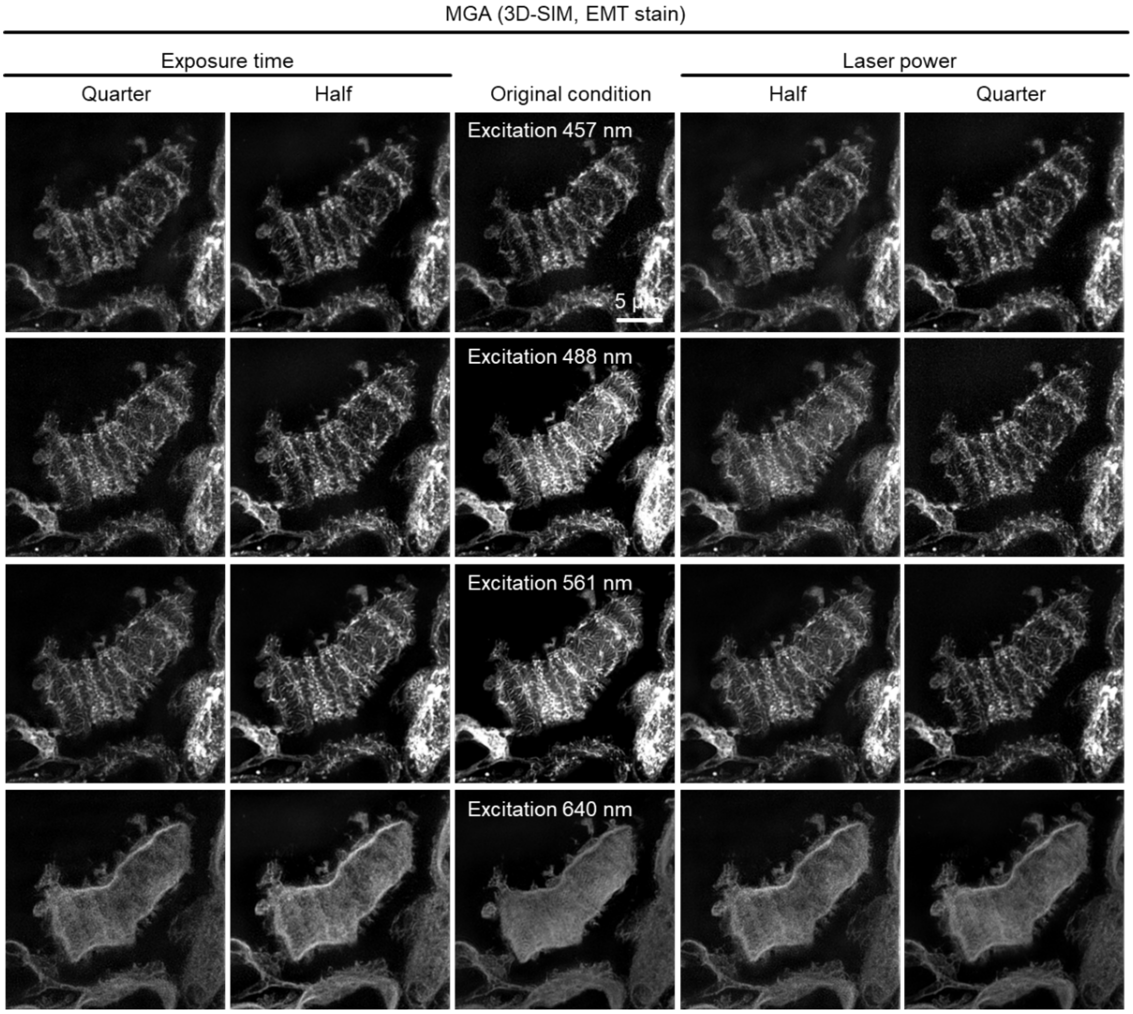

Figure S4. Optimization of scan parameters for EMT stained sections

Scan parameters of 3D-SIM were optimized to obtain best signal/noise ratio. Human kidney biopsy samples from patients diagnosed with minor glomerular abnormalities (MGA) were analyzed. Original condition indicates scan parameters shown in Table S5. Images obtained with various exposure times and laser powers are shown.

Supplementary Figure S5.

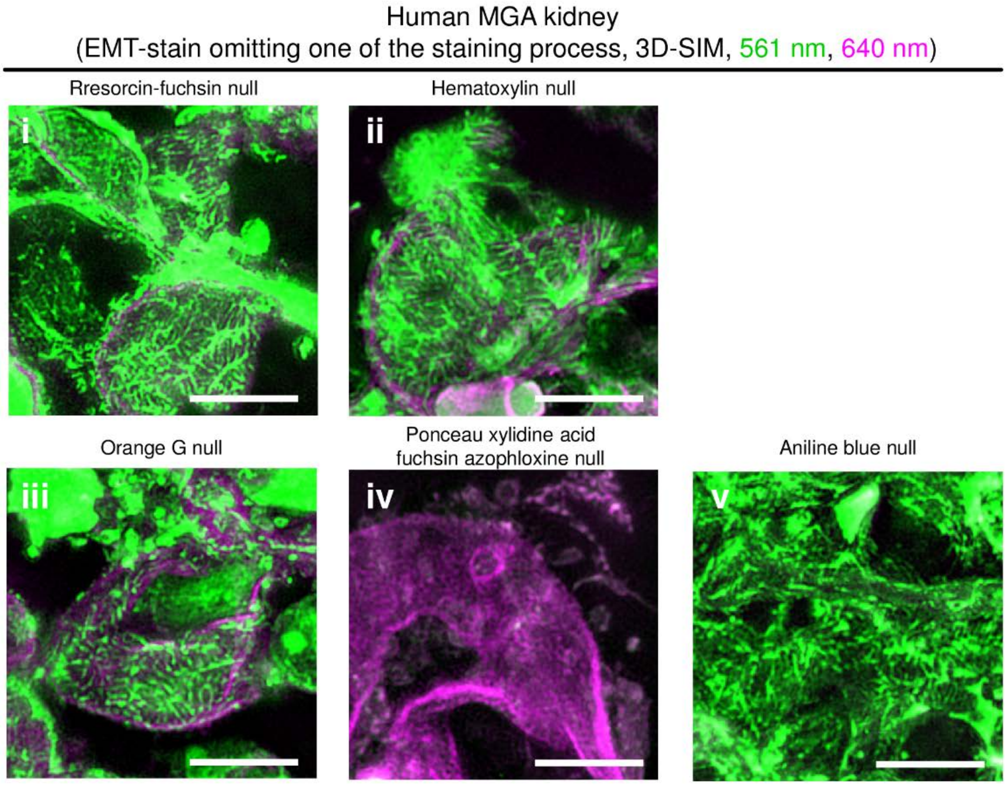

Figure S5. Human MGA kidney tissues were stained using modified EMT protocols

Human MGA kidney tissues were stained using modified EMT protocols that omitted (i) Maeda's resorcin-fuchisin (procedure 3 in Table S4), (ii) Wiegert's iron hematoxylin (procedure 8 in Table S4), (iii) orange G (procedure 13 in Table S4), (iv) ponceau xylidine acid fuchsin azophloxine (procedure 15 in Table S4), or (v) aniline blue (procedure 19 in Table S4). Representative 3D-SIM images of the capillary walls are shown (scale bars = 5 µm). Microstructures were visualized at 561 nm and 640 nm excitation and are pseudo-colored in green and magenta, respectively.

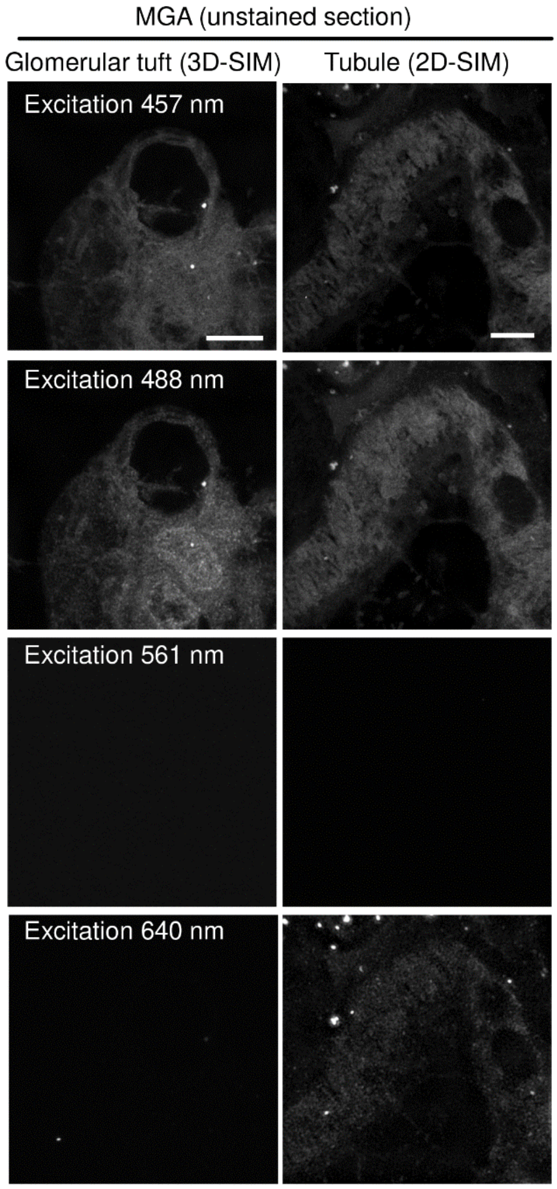

Figure S6. Micrographs obtained from unstained human kidney specimens

Unstained paraffin-embedded human kidney biopsy samples from patients with minor glomerular abnormalities (MGA) were observed using SIM. Representative micrographs are shown. (scale bars = 5 µm; N = 7 patients). All images were obtained under the settings for EMT-staining shown in Tables S5 and S6.

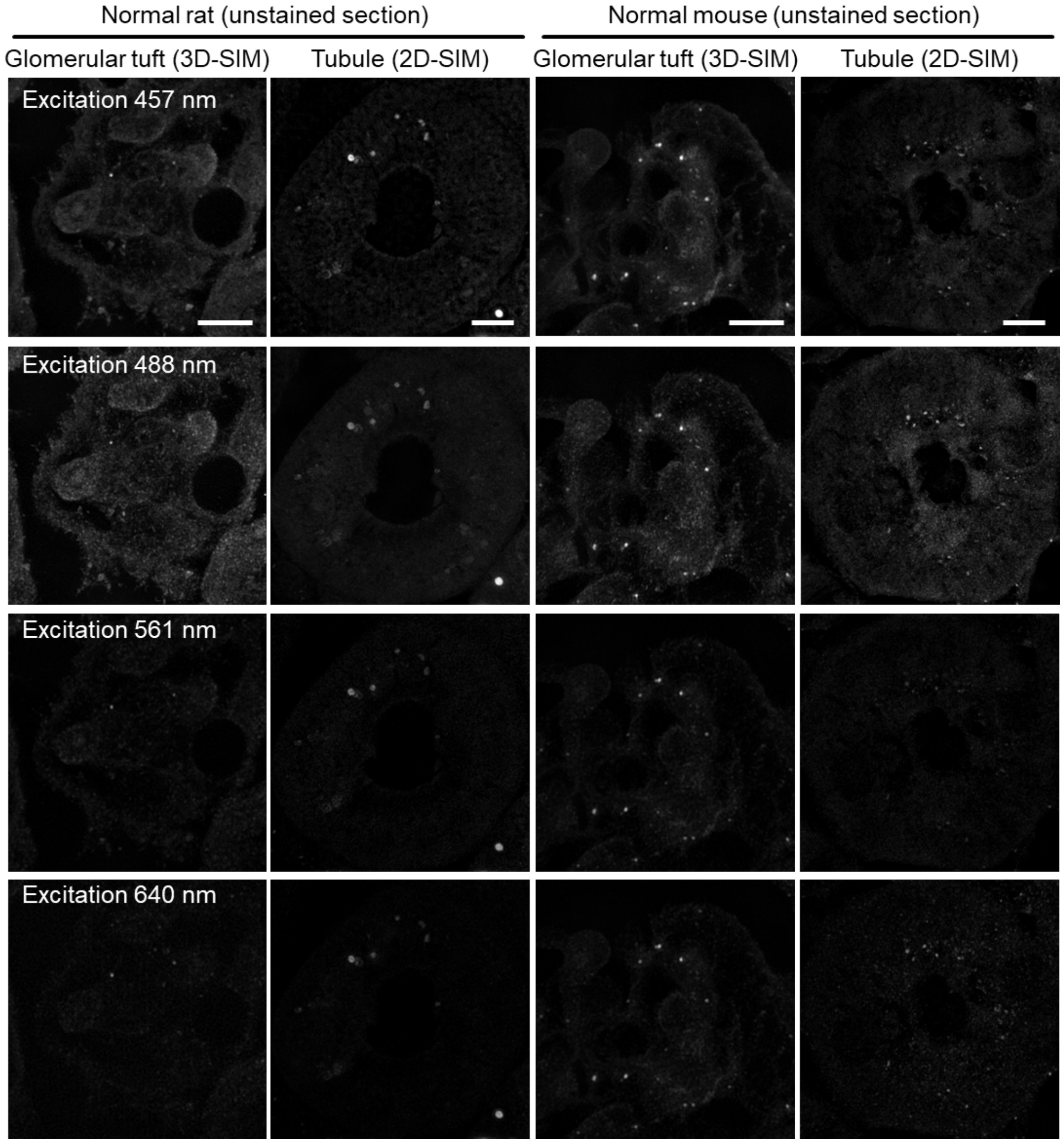

Figure S7. Micrographs obtained from unstained animal kidney specimens

Unstained paraffin-embedded normal rat and mouse kidney tissues were observed using SIM. Representative micrographs are shown. (scale bars = 5 µm). All images were obtained under the settings for EMT-staining shown in Tables S5 and S6.

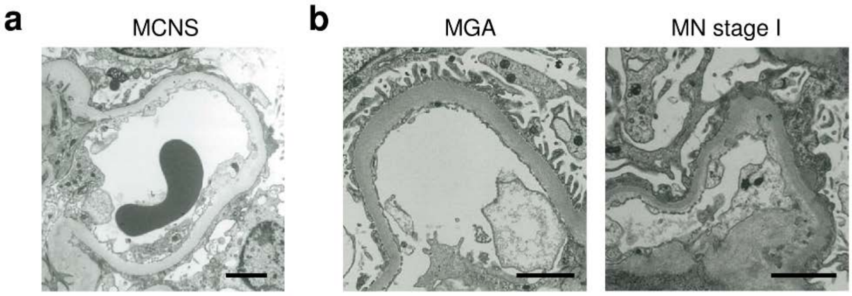

Figure S8. Electron micrographs of the patient specimens shown in Figures 6a and 2b

Glomerular electron micrographs of the patients shown in (a) Figure 6a and (b) Figure 2b (scale bars = 2 µm).

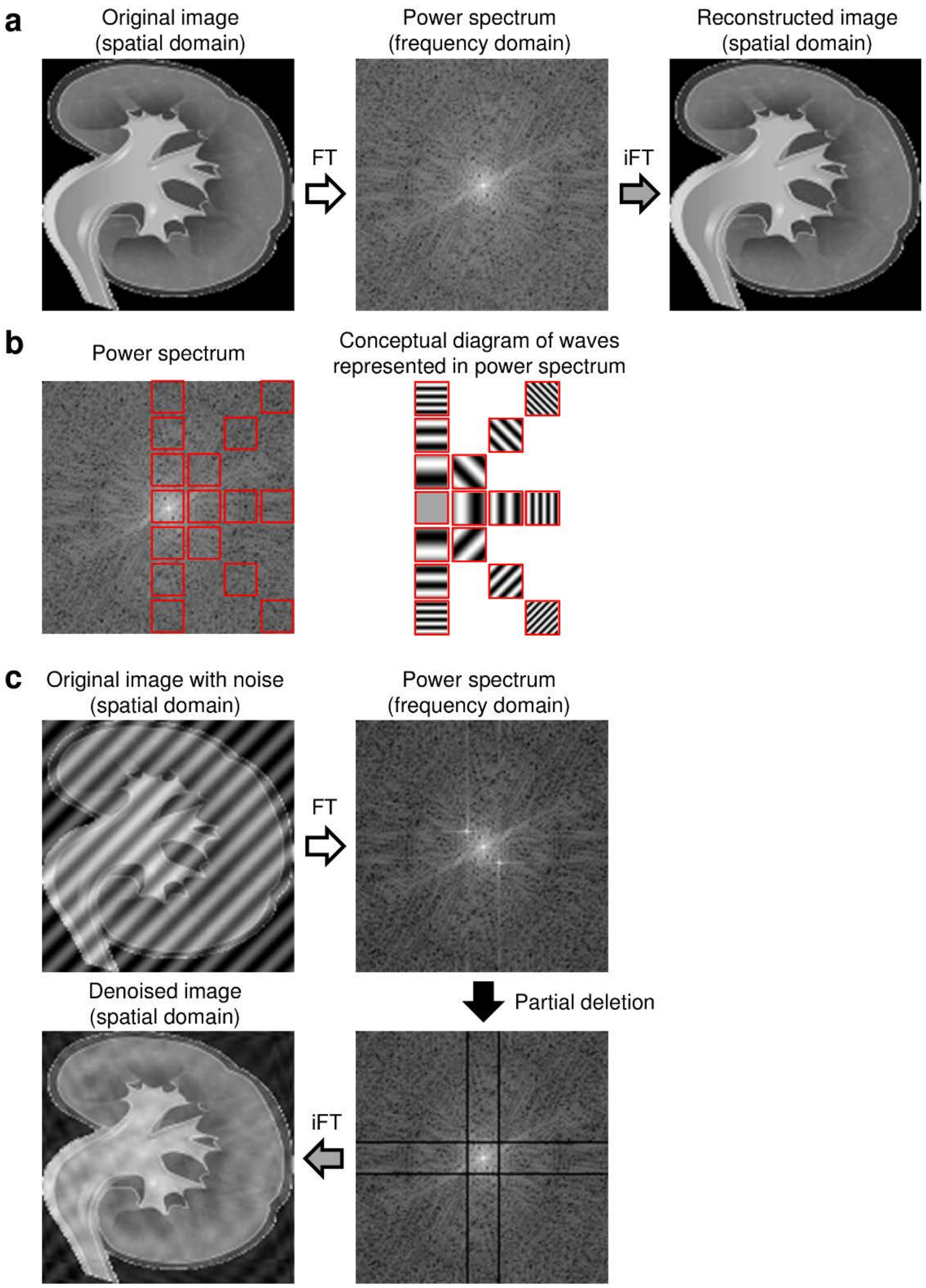

Figure S9. A concise illustration of Fourier transform

(a) Fourier transform (FT) decomposes a spatial domain of an image into its sine and cosine components (i.e. frequency domain). The power spectra (PS) are the result of FT and represents the image in the frequency domain, in which each point indicates the strength of a particular frequency contained in the original spatial domain image.

The PS can be reconverted into the original image through inverse FT (iFT). (b) The center portion of the PS represents the strength of the low frequency component, while the peripheral portion represents the strength of the high frequency component. (c) An image with periodic noise yields PS that contain signals representative of the noise, which can be removed to generate a denoised image in the spatial domain with iFT. These properties of FT indicate that information representing the noise in the spatial domain was included within the deleted portion of the frequency domain.

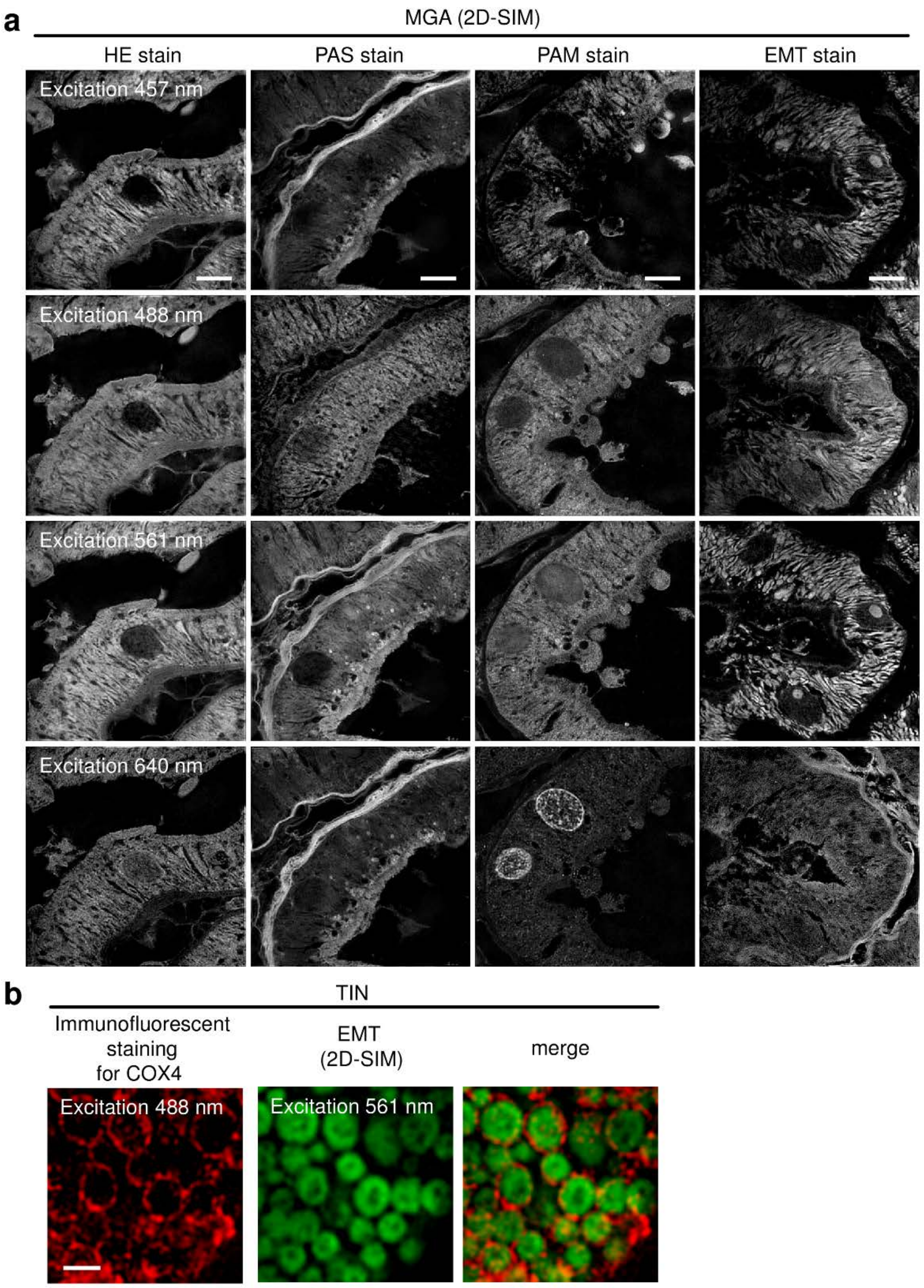

Figure S10. Mitochondrial structures were visualized in elastica Masson trichrome stained kidney sections

(a) Human kidney biopsy samples from patients diagnosed with minor glomerular abnormalities (MGA). Paraffin embedded 2-μm-thick kidney sections were stained with

HE, PAS, PAM, or EMT and observed with 2D-SIM. Representative 2D-SIM images of the tubulointerstitial area (bars = 5 µm) (N = 7 patients). (b) Kidney sections from patients diagnosed with tubulointerstitial nephritis (TIN) were stained with COX4 and then EMT (bar = 1 µm).

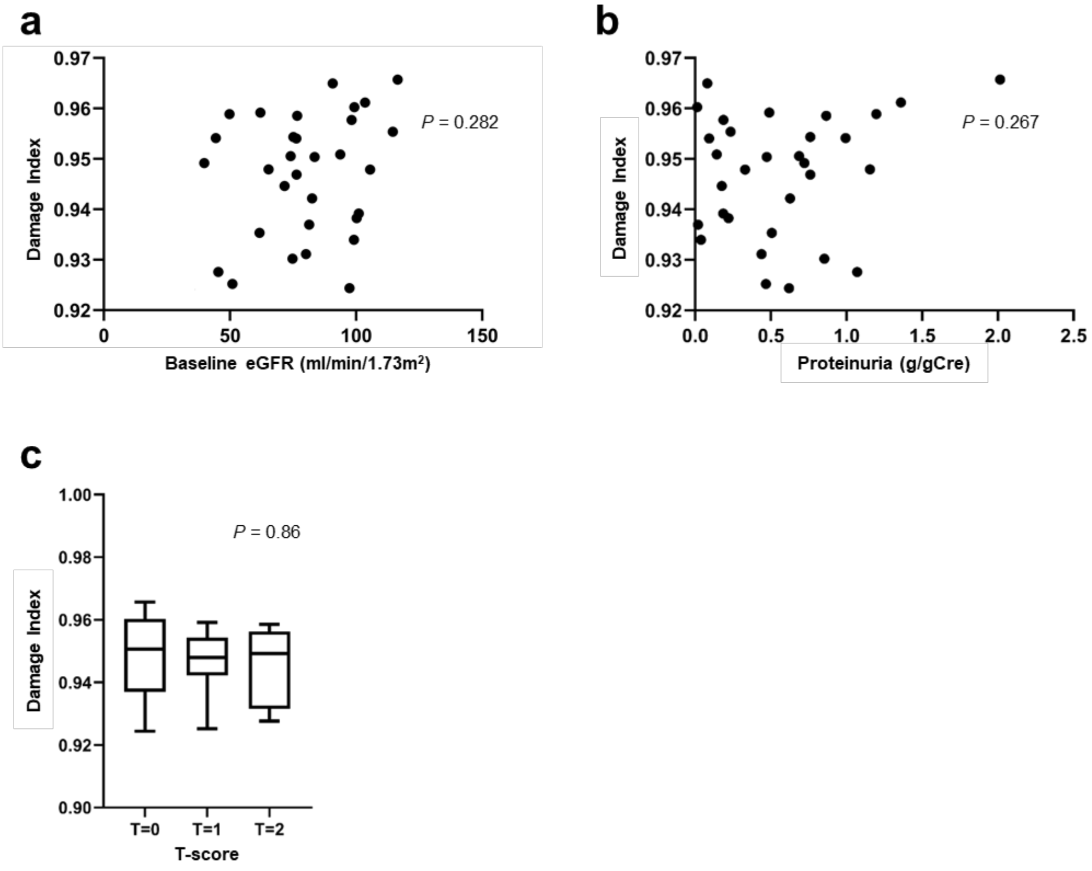

Figure S11. Mitochondrial damage index and clinical measurements

(a) The damage indices and baseline estimated glomerular filtration rate (eGFR) (R^2 = 0.0446; *P* = 0.282, robust linear regression). (b) The damage indices and proteinuria (R^2 = 0.0406; *P* = 0.267, robust linear regression). (c) The damage indices and the T-score of Oxford MEST-C score. (*P* = 0.86, ANOVA).
